# Revealing Spatial and Temporal Patterns of Cell Death, Glial Proliferation, and Blood-Brain Barrier Dysfunction Around Implanted Intracortical Neural Interfaces

**DOI:** 10.3389/fnins.2019.00493

**Published:** 2019-05-28

**Authors:** Steven M. Wellman, Lehong Li, Yalikun Yaxiaer, Ingrid McNamara, Takashi D. Y. Kozai

**Affiliations:** ^1^Department of Bioengineering, University of Pittsburgh, Pittsburgh, PA, United States; ^2^Center for the Neural Basis of Cognition, Pittsburgh, PA, United States; ^3^Eberly College of Science, Pennsylvania State University, University Park, PA, United States; ^4^Center for Neuroscience, University of Pittsburgh, Pittsburgh, PA, United States; ^5^McGowan Institute of Regenerative Medicine, University of Pittsburgh, Pittsburgh, PA, United States; ^6^NeuroTech Center, University of Pittsburgh Brain Institute, Pittsburgh, PA, United States

**Keywords:** oligodendrocytes, NG2 glia, pericytes, tissue-electrode interface, neurodegeneration, gliosis, glial cell division, inflammation

## Abstract

Improving the long-term performance of neural electrode interfaces requires overcoming severe biological reactions such as neuronal cell death, glial cell activation, and vascular damage in the presence of implanted intracortical devices. Past studies traditionally observe neurons, microglia, astrocytes, and blood-brain barrier (BBB) disruption around inserted microelectrode arrays. However, analysis of these factors alone yields poor correlation between tissue inflammation and device performance. Additionally, these studies often overlook significant biological responses that can occur during acute implantation injury. The current study employs additional histological markers that provide novel information about neglected tissue components—oligodendrocytes and their myelin structures, oligodendrocyte precursor cells, and BBB -associated pericytes—during the foreign body response to inserted devices at 1, 3, 7, and 28 days post-insertion. Our results reveal unique temporal and spatial patterns of neuronal and oligodendrocyte cell loss, axonal and myelin reorganization, glial cell reactivity, and pericyte deficiency both acutely and chronically around implanted devices. Furthermore, probing for immunohistochemical markers that highlight mechanisms of cell death or patterns of proliferation and differentiation have provided new insight into inflammatory tissue dynamics around implanted intracortical electrode arrays.

## Introduction

Advancements in neural interface technology have provided powerful tools for investigative neuroscience and clinical therapy involving neurodegenerative disease and neurological deficiencies (Schwartz et al., 2006; Kozai et al., 2015b; Iordanova et al., 2018; Michelson and Kozai, 2018; Michelson et al., 2018a; Stocking et al., 2019). Specifically, penetrating intracortical electrodes with high spatial resolution can record and stimulate individual neurons or neuronal populations locally within the brain (Buzsáki, 2004). However, due to a lack of fundamental knowledge regarding neural and glial circuits, neuromodulatory and stimulating devices experience high variability and unpredictability in their use (Sillay et al., 2010; Cheung et al., 2013; Gittis, 2018). Additionally, these probes are often debilitated by inflammatory tissue reactions that induce neural loss, impairing recording performances (Potter et al., 2012; Kozai et al., 2015c). Overwhelming biological responses to these inserted electrodes need to be addressed in order to improve the quality and robustness of chronically implanted intracortical arrays (Tresco and Winslow, 2011; Wellman et al., 2017, 2018; Wellman and Kozai, 2017). However, attempts at correlating a decline in signal quality with specific inflammatory events have previously proven difficult (Kozai et al., 2014c, 2015a; McCreery et al., 2016; Salatino et al., 2017; Michelson et al., 2018b).

Microglia are one of the first responders to probe insertion injury, polarizing and extending cellular processes within the first hour after implantation in an effort to encapsulate the probe (Kozai et al., 2012b, 2016b; Eles et al., 2017). During the course of implantation, reactive astrocytes mediate gliosis around the device by becoming hypertrophic, expanding and compacting their cellular membranes during scar formation (Szarowski et al., 2003; Polikov et al., 2005; Nolta et al., 2015). The inflammatory milieu secreted by activated glia proximal to the device has been suggested to be a major contributor to the characteristic loss of neurons, resulting in impaired recording performances (Kozai et al., 2015c). However, traditional immunohistochemical analyses involving neurons, microglia, and astrocytes around intracortical devices have not correlated well with recorded electrophysiology, in large part due to intra-animal variability at the electrode-tissue interface (Kozai et al., 2014c; McCreery et al., 2016; Michelson et al., 2018b). For example, tissue demonstrating high neural density and low glial scarring around implanted devices will still demonstrate reduced recording performance, or vice versa (Kozai et al., 2014c; Michelson et al., 2018b). Furthermore, neurons are not the only metabolically active cells susceptible to injury within the parenchyma, nor are microglia and astrocytes the only glial factors that contribute to inflammation during injury (Wellman and Kozai, 2017). Oligodendrocytes maintain important physiological roles mediating neuronal homeostasis via myelin ensheathment while oligodendrocyte precursors, notably NG2 glia, are responsible for replenishing depleted oligodendrocytes and participate in inflammation (Bradl and Lassmann, 2010). Additionally, perivascular pericytes are an essential component of the neurovascular unit and act to regulate blood-brain barrier (BBB) health and maintenance (Winkler et al., 2011). Each of these factors can influence neuronal viability during injury and, as of yet, their dynamics during electrode pathology are unknown.

Oligodendrocytes, a third glial component of the central nervous system, exist predominantly in white matter tracks alongside their myelin fibers but are also present at lower densities within the gray matter cortex (Tomassy et al., 2014). They provide trophic and mechanical support to neurons and promote signal propagation between neural circuits via myelin ensheathment [see review (Wellman et al., 2018)]. Secretion of neuronal growth factors requires oligodendrocytes and their precursors to maintain constant contact with neurons within the parenchyma (Du and Dreyfus, 2002). However, as energy-demanding cells, oligodendrocytes require high metabolic needs in order to produce and maintain the amount of myelin needed to support the central nervous system (McTigue and Tripathi, 2008; Bradl and Lassmann, 2010; Snaidero and Simons, 2017). As a result, oligodendrocytes and their precursors are highly susceptible to ischemic and hypoxic stress events (Dewar et al., 2003; McTigue and Tripathi, 2008). Since electrode insertion can induce stroke-like events, such as BBB disruption and loss of perfusion as well as glial cell activation, mechanical strain, and edema, oligodendrocytes and their precursors are vulnerable to the inflammation sustained from chronic microelectrode implantation (Du et al., 2017; Wellman and Kozai, 2017; Wellman et al., 2018). The only characterization of oligodendrocytes or myelin during electrode-induced inflammation was conducted by Winslow et al. (2010) where they presented evidence of chronic demyelination around an electrode array following 12 weeks of implantation (Winslow and Tresco, 2010). As of yet, oligodendrocyte and myelin pathology have not been thoroughly characterized around acute or chronically implanted devices.

Distributed ubiquitously throughout the central nervous system, oligodendrocyte precursor cells are essential in maintaining physiological support of neurons and act as a reservoir for myelinating oligodendrocytes in the event of oligodendrocyte loss or demyelinating injury (Levine et al., 2001). In regards to glial inflammation, they are known to respond to injury in a similar vein as microglia and react similarly to astrocytes through secretion of axon growth-inhibiting chondroitin sulfate proteoglycans, such as neural/glia antigen 2 (NG2)—thus, they are commonly referred to as NG2 glia (Nishiyama et al., 2005; Tan et al., 2005). NG2 glia also possess the ability to differentiate into reactive astrocytes under specific conditions of injury (Komitova et al., 2011). Using two-photon microscopy, the acute *in vivo* dynamics of microglia (Kozai et al., 2012b) and, more recently, NG2 glia (Wellman and Kozai, 2018) have been observed following microelectrode implantation, revealing a sequence of process extension and cell body migration in a specific spatiotemporal pattern of reactivity. However, beyond acute implantation, the distribution and proliferating patterns of NG2 glia have yet to be characterized around intracortical microelectrode arrays.

Pericytes are mural cells that interface directly between the BBB and parenchyma, mediating cross-talk between the brain and the peripheral circulation (Armulik et al., 2010). Another NG2-expressing cell within the brain, pericytes possess a variety of vascular homeostatic functions such as BBB maintenance, BBB repair, blood flow regulation, angiogenesis, as well as mesenchymal stem cell properties (Sweeney et al., 2016). Pericytes also facilitate neuroinflammatory reactions following injury and have been implicated as targets of interest in a variety of neurodegenerative diseases such as stroke, Alzheimer’s disease, multiple sclerosis, and more (Winkler et al., 2011). Many of these studies have correlated a reduction or loss in pericyte reactivity to occurrences of increased BBB permeability (Lindahl et al., 1997). Blood-brain barrier disruption has recently been recognized as a significant factor of inflammation induced by intracortical electrodes and a potential perpetrator of reduced device performance (Kozai et al., 2010, 2015c). Further investigation of pericyte behavior and reactivity to injury is required to understand how they fit into these sequences of inflammatory events.

In order to fill the gaps in knowledge surrounding these novel cell types of interest, additional immunohistochemical markers were employed (oligodendrocytes, NG2 glia, pericytes) alongside traditional stains (neurons, microglia, and astrocytes) to observe spatiotemporal dynamics following intracortical microelectrode implantation. Markers for cellular apoptosis and proliferation were also used to observe mechanisms of cell death or patterns of division, respectively. Probes were implanted acutely for 1, 3, and 7 days as well as chronically for 28 days post-insertion. These analyses answer critical questions about unknown or understudied parenchymal components during electrode-induced inflammation that can guide future studies to determine mechanistic causes of signal quality degradation or assist in the development of novel therapies to improve device performances.

## Materials and Methods

### Surgical Probe Implantation

Implantation of microelectrode arrays were performed as described previously (Kozai et al., 2014c, 2016c). Prior to surgery, all electrode arrays, surgical tools, and surgical supplies were sterilized using ethylene oxide for 12 h. Single shank non-functional Michigan-style microelectrodes (A16-3 mm-100-703-CM15) were implanted into the left primary monocular visual cortex (V1m) of 8-week old C57BL/6J male mice (Jackson Laboratory, Bar Harbor, ME). Mice were anesthetized using a 7 mg/kg xylazine and 75 mg/kg ketamine cocktail injected intraperitoneally and mounted onto a stereotaxic frame. Eye ointment was administered to keep the eye moisturized and an O_2_ line was installed for oxygen delivery. Betadine and alcohol scrubs were administered to sterilize the surgical area prior to removing the scalp and connective tissue from the surface of the skull. A thin layer of Vetbond (3 M) was applied to dry the surface of the skull and provide enhanced adhesion between the skull and dental cement. Three bone screws were drilled into the bone over both motor cortices and the contralateral visual cortex to help secure the headcap. A 1 mm drill-sized craniotomy was formed at 1 mm anterior to lambda and 1.5 mm lateral from the midline using a high-speed dental drill. Saline was periodically administered to prevent overheating of the brain surface. After the craniotomy was removed and brain exposed, gelfoam was used to prevent drying out of the brain prior to implantation. The single shank device was carefully inserted using a stereotaxic manipulator at a speed of ∼2 mm/s until the last electrode contact site was below the surface (∼1600 μm from the pial surface). The inserted probe was sealed using a silicone elastomer (Kwik-sil) and a headcap was secured with UV-curable dental cement (Henry Schein, Melville, NY, United States). Non-steroidal anti-inflammatory ketofen was administered at 5 mg/kg for 2 days post-operatively.

### Endpoint Histology

Mice were sacrificed and perfused according to University of Pittsburgh IACUC approved methods at days 1, 3, 7, and 28 days post-insertion (*n* = 3 per time point). Prior to perfusion, a 7 mg/kg xylazine and 75 mg/kg ketamine cocktail was administered to deeply anesthetize each mouse and a toe-pinch test was used to determine a proper plane of anesthesia. Mice were transcardially perfused (pump pressure: 80–100 mm Hg) using 100 mL of a warm phosphate buffered saline (PBS) flush followed by 100 mL of 4% paraformaldehyde. Following perfusion, mice were decapitated and heads were post-fixed in 4% paraformaldehyde at 4°C overnight. Brains were then removed from the skull and soaked sequentially in 15 and 30% sucrose baths at 4°C for 24 h each. Following this, brains were carefully separated from the device and headcap and soaked in 30% sucrose for another 12–24 h. After sucrose equilibration, brains were blocked and frozen in a 2:1 20% sucrose in PBS:optimal cutting temperature compound (Tissue-Tek, Miles Inc., Elkhart, IN, United States). Samples were sectioned horizontally at a 25 μm slice thickness onto glass slides using a cryostat (Leica Biosystems, Wetzlar, Germany).

In order to minimize variability due to layer-specific differences in cell distribution, sections between a depth of 400–800 μm (layer IV-V) were chosen for immunohistochemical analysis. Additionally, sections free of extraneous tissue holes that could arise during probe extraction, perfusion, or sectioning were chosen for analysis. Prior to staining, sections were re-hydrated with 1× PBS for 30 min. Slides were incubated in 0.01 M sodium citrate buffer (for antigen retrieval) for 30 min at 60°C followed by incubation in a peroxidase blocking solution (10% v/v methanol and 3% v/v hydrogen peroxide) for 20 min on a table shaker at room temperature. Sections were then permeabilized using a solution of 1% triton X-100 with 10% donkey serum in PBS for 30 min at RT. Lastly, sections were blocked with donkey anti-mouse IgG fragment (Fab) or 647 conjugated anti-mouse IgG fragment (Fab) for 2 h at 1:13 or 1:16 dilution at RT.

Following 1× PBS rinses (8 × 4 min), sections were incubated with a primary antibody solution consisting of 10% donkey serum, 1% triton X-100, and antibodies listed in Table 1 overnight at 4°C. Sections were then rinsed with 1× PBS (3 × 5 min) before being incubated with a secondary antibody solution in 1× PBS (donkey anti-mouse 405, Abcam, and donkey anti-goat 568, Abcam, Cambridge, United Kingdom; donkey anti-mouse IgG 488, Thermo Fisher Scientific, donkey anti-rabbit IgG 568, Thermofisher, and streptavidin, Alexa Fluor 488 conjugate, Thermo Fisher Scientific, Waltham, MA, United States; and donkey anti-chicken IgY 647, Sigma-Aldrich, St. Louis, MO, United States) diluted at 1:500 for 2 h at RT. Following 3× PBS washes for 5 min, sections were incubated with 1:1000 Hoechst 33342 (Invitrogen) for 10 min and then washed one final time with 1× PBS (3 × 5 min) before being coverslipped using Fluoromount-G (Southern Biotech, Birmingham, AL, United States) and sealed with fingernail polish. Samples were imaged at 16-bit (635.9 × 635.9 μm, 1024 × 1024 pixels on FV10-ASW Viewer V4.2) using a confocal microscope (FluoView 1000, Olympus, Inc., Tokyo, Japan) with a 20× oil-immersive objective lens at the Center for Biologic Imaging at the University of Pittsburgh.

**Table 1:** List of primary antibodies used for immunohistological staining.

Primary antibody	Marker	Supplier	Species	Dilution
Anti-NeuN	Neurons	Sigma-Aldrich (MAB377B)	Ms	1:200
Anti-Iba-1	Microglia	Sigma-Aldrich (MABN92), Fisher (NC9288364)	Ms, Rb	1:200 (Ms), 1:500 (Rb)
Anti-GFAP	Astrocytes	Abcam (AB4674)	Ck	1:500
Anti-CC1	Mature oligodendrocytes	Abcam (AB7474)	Rb	1:400
Anti-Olig2	Immature oligodendrocytes	Sigma (MABN50)	Ms	1:100
Anti-NG2	NG2 glia, pericytes	Sigma (AB5320)	Rb	1:200
Anti-NF-200	Neurofilament	Sigma (MAB5256)	Ms	1:250
Anti-MBP	Myelin basic protein	Abcam (AB7349)	Rt	1:100
Anti-PDGFR-β	Pericytes	R&D Systems (AF1042)	Gt	1:100
Anti-Lectin	Blood vessels	Vector Lab (B-1175)	Tomato	1:250
Anti-Ki67	Proliferation marker Ki67	BD biosciences (550609)	Ms	1:50
Anti-Activated caspase 3	Activated caspase 3	Cell signal (9661S)	Rb	1:400


### Data Analysis

Intensity analyses of immunohistochemical stains (NF-200/MBP/Iba-1/NG2/GFAP) were performed radially around the probe hole using a custom script (INTENSITY Analyzer) written in MATLAB (Mathworks, Natick, MA, United States) developed previously (Kozai et al., 2014b). Bins spaced 10 μm apart up to 300 μm away from the probe hole were generated as concentric rings around the probe hole and the average grayscale intensity was calculated for all pixels above a threshold determined by the intensity of the background noise within each bin. Intensity data was normalized using data from the four corners of each image representing tissue 300 μm or more away from the insertion site. Data was averaged over all animals per time point and per bin and reported as mean ± standard error as a function of distance from the insertion site. Additionally, bar graphs of intensity data averaged over 50 μm away from the probe hole were generated in a separate MATLAB script and reported as mean ± standard error for each time point.

For cell counting (NeuN/CC1/Iba-1/NG2/GFAP/PDGFR-β/Caspase-3/Ki67), the MATLAB script was modified to generate bins spaced 50 μm apart up to 300 μm away from the probe hole. For neuronal and oligodendrocyte density, tissue area was calculated within each bin after excluding tissue “holes” and the density was calculated as total cell count divided by tissue area per bin. Tissue holes were determined by any pixel values that were less than 1 by optimizing image offset during image acquisition. For co-localization analysis, two or more immunohistochemical markers (NeuN/Caspase-3, CC1/Caspase-3, Iba-1/Ki67, NG2/Ki67, GFAP/Ki67, GFAP/Olig2) were merged and quantified in ImageJ (Schindelin et al., 2012). All cell counts were performed against a DAPI stain to confirm the presence of cell nuclei. Similar to the intensity analysis, data was averaged over all animals per time point per bin and reported as mean ± standard error as a function of distance from the probe hole.

### Statistics

A two-way ANOVA followed by a *post hoc* Tukey HSD test was used on cell density and fluorescence intensity analysis generated per animal per time point (*n* = 3) to evaluate significant differences between time points of implantation. An unequal variance two-tailed *t*-test was used to determine significant differences between co-localized cell counts at different time points. A *p*-value < 0.05 was chosen for significance. Instances of no significance does not mean there is no actual difference between groups, but rather that there is no observable significance given the group size. However, instances in which significant differences are reported demonstrate the robustness of those differences given appropriately applied statistical analysis.

## Results

### Impact on Neuronal Viability and Axonal Structures Following Microelectrode Implantation

**FIGURE 1:**
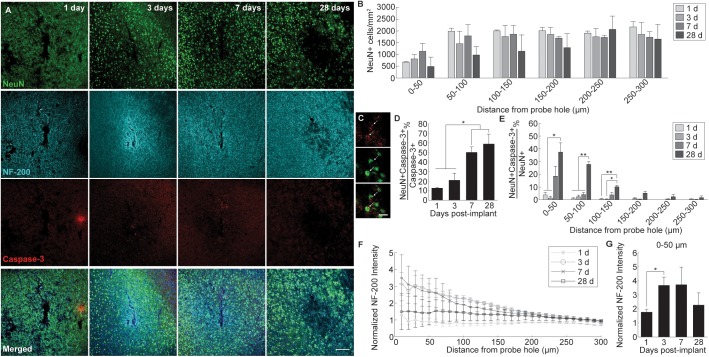
Evaluation of neuronal cell death and axonal preservation around an implanted microelectrode array. Microelectrode implantation induces progressive neuronal loss and degeneration as well as axonal dysfunctional within close proximity of the electrode interface. **(A)** Representative immunohistochemical stain for neurons (NeuN), axons (NF-200), and caspase-3 at 1, 3, 7, and 28 days post-insertion. Scale bar = 100 μm. **(B)** NeuN+ cell counts within 50 μm bins up to 300 μm away from the probe hole demonstrating reduced neural density near the site of probe insertion. Notably, neuronal density is most disrupted within 0–100 μm from the probe hole at each time point. **(C)** Magnified images of Caspase-3+ staining (top), NeuN+ cells (middle), and co-labeled NeuN+Caspase-3+ cells (bottom). White arrows denote co-localized cells. Scale bar = 20 μm. **(D)** Percent of NeuN+Caspase-3+ cells over total Caspase-3+ cells within 300 μm from the probe hole. **(E)** Increases in percent of NeuN+Capase-3+ cells over total NeuN+ cells within 50 micron bins up to 300 μm away from the probe hole demonstrate significant neurodegeneration near the site of implantation. **(F)** Axonal reorganization is presented as normalized NF-200 intensity within 10 μm bins up to 300 μm away from the probe hole. **(G)** Normalized NF-200 intensity averaged over the first 50 μm from the probe hole highlights significant temporal differences in axonal integrity near the site of insertion. Pattern of axonal intensity matches fluctuations in neuronal density over time within 0–50 μm distance from probe hole. ^∗^ indicates *p* < 0.05. ^∗∗^ indicates *p* < 0.01.

Neuronal cell distribution and axonal integrity were evaluated using markers specific to neuronal nuclei (NeuN) and neurofilament protein (NF-200), respectively, at 1, 3, 7, and 28 days following insertion (Figure 1A). Additionally, activated caspase-3 was co-stained to label for cellular degeneration. At regions distal to the probe implant site, NeuN+ cell density was consistent with previously reported values (∼1500 NeuN+ cells/mm^2^ at 200–300 μm away from probe hole) affirming the robustness of the histology (Golabchi et al., 2018). While no significant differences were noted, NeuN+ cell density counts revealed a biphasic pattern of reduced neural density occurring at 1 and 28 days and increased neural density at 3 and 7 days post-insertion (Figure 1B). Neuronal loss occurred most markedly within 0–50 μm away from the probe hole. To determine mechanistic causes of neuronal loss around the implanted microelectrode array, activated caspase-3 staining was used to observe instances of neurodegeneration (Figure 1C). Of the total caspase-3+ cell population observed within 300 μm from the site of device insertion, NeuN+ caspase-3+ cells steadily increased over time from 1 to 28 days post-insertion, with a significant increase in neurodegeneration present at 7 (50 ± 5.7% NeuN+Casp3+/NeuN+) and 28 (58.7 ± 10.3% NeuN+Casp3+/Casp3+) days post-insertion (*p* < 0.05) (Figure 1D and Supplementary Table S1). To determine the spatial distribution of neurodegeneration, NeuN+ caspase-3+ cells were taken as a proportion of total NeuN+ cells within 50 μm bins up to 300 μm from the probe hole (Figure 1E and Supplementary Table S2). Within 150 μm from the site of insertion at 28 days post-implantation, the percentage of caspase-3+ cells within the NeuN+ population was significantly increased compared to 1 and 3 days post-insertion (*p* < 0.05). At 100–150 μm away from the probe hole, there was a significant increase at 28 days compared to 1 and 3 days post-insertion (*p* < 0.01). Analysis of axonal structures around the microelectrode array revealed an increase in neurofilament intensity beginning at 3 and 7 days post-insertion before decreasing at 28 days post-insertion (Figure 1F). Neurofilament expression was highest with proximity to the implanted device, decreasing to baseline control values further from the site of implantation. Average NF-200 intensity within the first 50 μm from the device demonstrated a significant increase from 1 to 3 days post-insertion (*p* < 0.05) (Figure 1G and Supplementary Table S3).

### Impact on Oligodendrocyte Viability and Myelination Following Microelectrode Implantation

**FIGURE 2:**
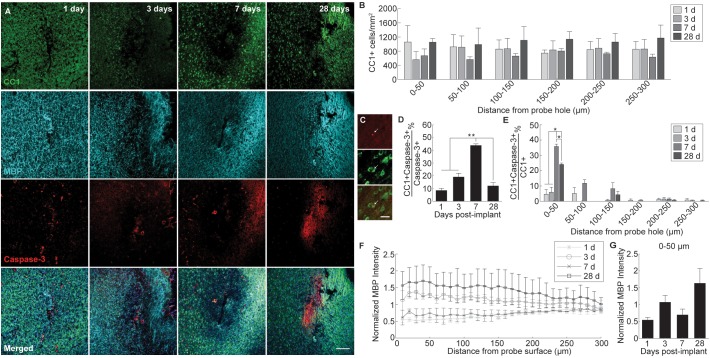
Evaluation of oligodendrocyte cell death and myelin preservation around an implanted microelectrode array. Acute microelectrode implantation compromises oligodendrocyte viability and function, preceding abnormal myelin expression and reorganization around the electrode interface. **(A)** Representative immunohistochemical stain for mature oligodendrocytes (CC1), myelin (MBP), and caspase-3 at 1, 3, 7, and 28 days post-insertion. Scale bar = 100 μm. **(B)** CC1+ cell counts within 50 μm bins up to 300 μm away from the probe hole indicating fluctuations in oligodendrocyte density around the implanted probe. Compared to neurons, oligodendrocyte densities were robust from 1 to 28 days post-insertion, fluctuating between time points but remaining relatively stable over time. **(C)** Magnified images of Caspase-3+ staining (top), CC1+ cells (middle), and co-labeled CC1+Caspase-3+ cells (bottom). White arrows denote co-localized cells. Scale bar = 20 μm. **(D)** Oligodendrocyte degeneration is noted as a percent of CC1+Caspase-3+ cells over total Caspase-3+ cells within 300 μm from the probe hole. *Note*: Peak oligodendrocyte degeneration preceeds peak neuronal degeneration. **(E)** Percent of CC1+Capase-3+ cells over total CC1+ cells within 50 micron bins up to 300 μm away from the probe hole. **(F)** Normalized MBP intensity within 10 μm bins up to 300 μm away from the probe hole. **(G)** Normalized MBP intensity averaged over the first 50 μm from the probe hole demonstrates an increase in myelination around the site of insertion at chronic timepoints. An increase in myelination at 28 days post-insertion coincides with a decrease in oligodendrocyte degeneration, indicating a potential reparative effect. ^∗^ indicates *p* < 0.05. ^∗∗^ indicates *p* < 0.01.

Alterations to oligodendrocyte distribution and myelin organization were evaluated using markers for mature oligodendrocytes (CC1) and myelin basic protein (MBP), respectively, at 1, 3, 7, and 28 days following device implantation (Figure 2A). Similar to the neuronal analysis, activated caspase-3 was used to determine the prevalence of induced cellular degeneration in oligodendrocytes. Normalized CC1+ cell density revealed a pattern of oligodendrocyte loss occurring at 3 and 7 days post-insertion (Figure 2B). By 28 days post-insertion, an increase in oligodendrocyte population could be observed, however, there was no significant difference reported (Figure 2B). Reductions in oligodendrocyte density occurred most heavily within 0–50 μm from the site of insertion, implying decreases in oligodendrocyte viability are in response to the implanted device. The percentage of total caspase-3+ cells that were CC1+ within a 300 μm region around the device (CC1+Casp3+/Casp3+) revealed that apoptosis-induced oligodendrocyte cell death occurs most markedly at 7 days post-insertion (43.7 ± 1.3%), significantly increased from 1 (8.45 ± 1.6% CC1+Casp3+/Casp3+) and 3 days (19 ± 3%) following implantation (*p* < 0.01) (Figure 2C,D and Supplementary Table S4). The proportion of caspase-3+ cells that were CC1+ significantly decreased from 7 to 28 (12 ± 2.6%) days following insertion (*p* < 0.01). Additionally, the percentage of caspase-3+ cells within the total CC1+ population peaked significantly at 7 days post-insertion, specifically within 0–50 μm away from the probe hole indicative of a temporal pattern of oligodendrocyte degeneration around the implanted device (Figure 2D and Supplementary Table S5). Staining for MBP expression demonstrated a decrease in myelin content proximal to the device at 1 and 7 days post-insertion and an overall increase in myelin intensity at 28 days following implantation (Figure 2E). Average MBP intensity within the first 50 μm from the device emphasizes this pattern, with MBP expression increasing 1.5–2 fold by 28 days post-insertion (Figure 2F). Notably, instead of reduced or deplete regions of myelin expected of myelin degeneration, MBP appeared with increased intensity near the site of implantation, similar to neurofilament, indicative of an upregulation in myelin basic protein that would occur during reorganization of myelinated axons or remyelination of demyelinated axons following injury. However, no significant differences between each time point were observed.

### Pattern of Glial Cell Immunoreactivity and Proliferation Around Implanted Microelectrode Arrays

**FIGURE 3:**
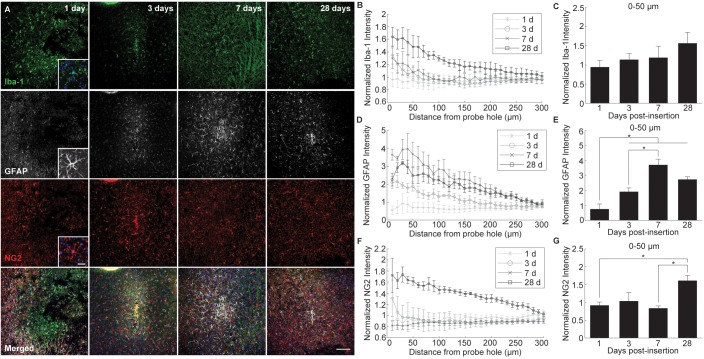
Evaluation of glial cell activation around an implanted microelectrode array. Microelectrode implantation induces distinct spatial and temporal patterns of microglia, astrocyte, and NG2 glia reactivity. **(A)** Representative immunohistochemical stain for microglia (Iba-1), NG2 glia (NG2), and astrocytes (GFAP) at 1, 3, 7, and 28 days post-insertion. Scale bar = 100 μm. Inserts show magnified view of Iba-1+, GFAP+, and NG2+ cells with nuclei stain. Scale bar = 20 μm. **(B)** Normalized Iba-1 intensity within 10 μm bins up to 300 μm away from the probe hole. **(C)** Linear increases in microglia reactivity around the implanted device is depicted as normalized Iba-1 intensity averaged over the first 50 μm from the probe hole. **(D)** Normalized GFAP intensity within 10 μm bins up to 300 μm away from the probe hole. **(E)** Normalized GFAP intensity averaged over the first 50 μm from the probe hole demonstrates temporal patterns in astrocyte scar formation around the implanted device. **(F)** Normalized NG2 intensity within 10 μm bins up to 300 μm away from the probe hole. **(G)** Normalized NG2 intensity averaged over the first 50 μm from the probe hole demonstrates a significant increase in NG2 expression at chronic timepoints. NG2 intensity decreases at 7 days when GFAP intensity peaks. Additionally, NG2 intensity peaks at 28 days as oligodendrocyte degeneration decreases and myelination increases. ^∗^ indicates *p* < 0.05.

Glial reactivity to implanted intracortical devices was evaluated by staining for histological markers specific for microglia (Iba-1), astrocytes (GFAP), and NG2 glia (NG2) at 1, 3, 7, and 28 days post-insertion (Figure 3A). Iba-1 immunoreactivity was highest proximal to the implant for all time points, decreasing steadily to normalized control levels with distance from the probe hole (Figure 3B). When observing directly at the 50 μm region at tissue-device interface, Iba-1 expression increased steadily over time; however, there was no significant difference in fluorescence intensity (*p* < 0.05) (Figure 3C). Beginning 3 days after implantation, GFAP fluorescence intensity increased 2–4 fold with proximity to the probe hole showing preferential expression around the site of device implantation (Figure 3D). GFAP expression was minimal at 1 day post-insertion and the average GFAP fluorescence was significantly increased within the 50 μm region surrounding the device at 3, 7, and 28 days post-implantation (*p* < 0.05) (Figure 3E and Supplementary Table S6). GFAP intensities reached a maximum at 7 days, and was significantly increased from expression levels at 3 days post-insertion (*p* < 0.05). Interestingly, NG2 expression increased adjacent to the implant at 3 days post-insertion; however, it remained reduced at 1 and 7 days following implantation (Figure 3F). Similar to Iba-1 expression, NG2 expression was highest at 28 days post-insertion and was significantly higher within 0–50 μm compared to NG2 fluorescence intensity at 1 and 7 days post-insertion (*p* < 0.05) (Figure 3G and Supplementary Table S7).

**FIGURE 4:**
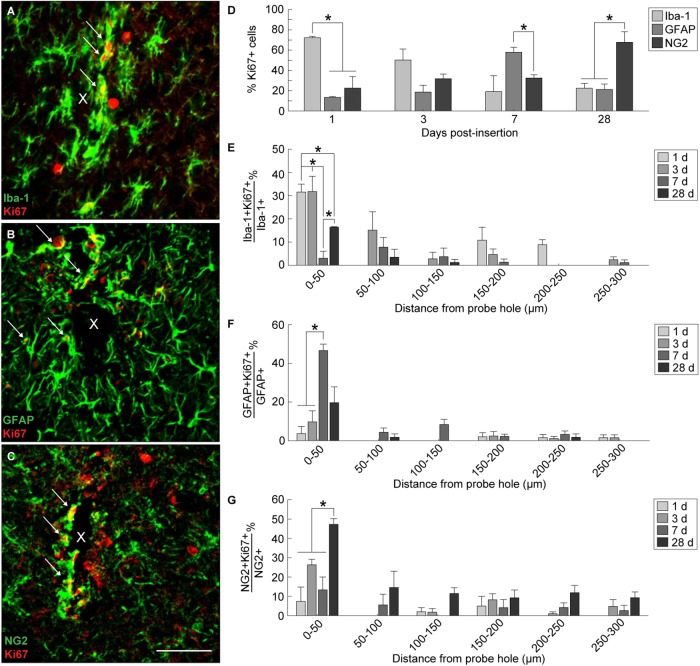
Tracking proliferation of glial cells around an implanted microelectrode array. Microglia, astrocytes, and NG2 glia demonstrate distinct temporal patterns of proliferation following device insertion. Additionally, glial proliferation occurs most prominently at the site of insertion (0–50 μm distance from probe hole). Representative immunohistochemical stain for Iba-1+ **(A)**, GFAP+ **(B)**, and NG2+ **(C)** cells that express the Ki67+ proliferation marker. White arrows denote co-localized cells and indicate glial proliferation. Center of the probe hole is denoted by a white “x.” Scale bar = 50 μm. **(D)** Percent of Ki67+ cells within 300 μm from the probe hole which are Iba-1+, GFAP+, or NG2+ at 1, 3, 7, and 28 days post-insertion reveal a distinct temporal pattern of proliferation. **(E)** Percent of Iba-1+Ki67+ cells over total Iba-1+ cells within 50 μm bins up to 300 μm away from the probe hole demonstrate early microglia proliferation around the implanted device. **(F)** Percent of GFAP+Ki67+ cells over total GFAP+ cells within 50 μm bins up to 300 μm away from the probe hole reveal astrocytes as the predominantly dividing glia following microglia. **(G)** The percent of NG2+Ki67+ cells over total NG2+ cells within 50 μm bins up to 300 μm away from the probe hole reveals chronic proliferation of NG2 glia. NG2 proliferation decreases at 7 days when astrocyte proliferation peaks. Additionally, NG2 proliferation peaks at 28 days as oligodendrocyte degeneration decreases and myelination increases. ^∗^ indicates *p* < 0.05.

In order to evaluate the extent of dividing glia around the implanted device, Ki67, a marker for cellular proliferation, was co-stained along with Iba-1, GFAP, and NG2 (Figure 4A–C). Co-localization of Ki67+ cells with Iba-1+, GFAP+, and NG2+ cells revealed a distinct temporal pattern of division within 300 μm from the site of implantation (Figure 4D and Supplementary Table S8). At 1 day post-insertion, Iba-1+Ki67+ cells consisted of a significant proportion of Ki67+ cells (72.14 ± 1.5%) compared to GFAP+Ki67+ (13.3 ± 0.8%) and NG2+Ki67+ cells (22.5 ± 11.4%) (*p* < 0.05). By 3 days post-insertion, Iba-1+ cells remained the most co-localized with Ki67+ cells (50.14 ± 11%) compared to GFAP+ (18.6 ± 6.7%) and NG2+ cells (31.7 ± 4.6%); however, there was no significant difference between the three populations. At 7 days post-insertion, GFAP+ cells comprised a significant proportion of Ki67+ cells (57.9 ± 4.8%) compared to NG2+ cells (32.5 ± 3.1%) (*p* < 0.05) but not compared to Iba-1+ cells (19 ± 15.6%). By 28 days post-insertion, NG2+ cells were co-localized significantly with Ki67+ cells (67.6 ± 10.5%) compared to Iba-1+ (22.5 ± 4.7%)and GFAP+ cells (21.2 ± 5.3%) (*p* < 0.05). To observe potential spatial patterns in glial proliferation, co-localized Ki67+ glial cells were taken as a proportion of total Iba-1+, GFAP+, and NG2+ cells counted within 50 μm bins up to 300 μm away from the probe hole. Spatial analysis determined that, for any given time point, the majority of proliferating glia preferentially resided with close proximity to the device with the highest percentage of Ki67+ glia appearing within 50 μm from the probe hole (Figure 4E–G). Of the total Iba-1+ population, the proportion of Iba-1+Ki67+ cells was significantly increased at 1 and 3 days post-insertion (*p* < 0.05) (Figure 4E and Supplementary Table S9). The percentage of Iba-1+Ki67+ cells was reduced at 7 days before significantly increasing by 28 days post-insertion (*p* < 0.05). Within the GFAP+ population, the percentage of Ki67+GFAP+ cells within 50 μm from the site of insertion peaked at 7 days post-insertion, significantly increased from 1 and 3 days after implantation (*p* < 0.01) (Figure 4F and Supplementary Table S10). Finally, the percentage of Ki67+NG2+ cells within 50 μm from the site of insertion was significantly increased at 28 days compared to 1, 3, and 7 days post-insertion (Figure 4G and Supplementary Table S11).

### Appearance of Subpopulation of Olig2+Reactive Astrocytes Around Implanted Microelectrode Arrays

**FIGURE 5:**
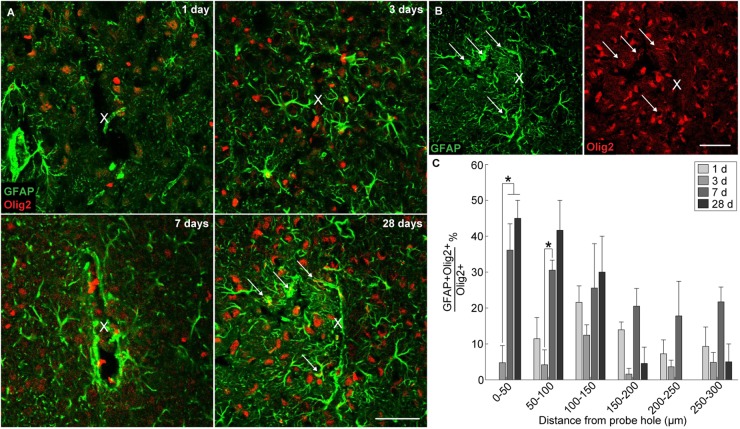
Generation of reactive astrocytes from oligodendrocyte lineage cells around an implanted microelectrode array. Following microelectrode implantation, astrocytes expressed the oligodendrocyte transcription factor Olig2, which may implicate oligodendrocyte lineage cells as a source of astrocytes following injury. **(A)** Representative immunohistochemical stains for GFAP (green) and Olig2 (red) at 1, 3, 7, and 28 days post-insertion revealing a distinct subpopulation of Olig2+GFAP+ astrocytes. White arrows denote co-localized cells. Center of the probe hole is denoted by a white “x.” Scale bar = 50 μm. **(B)** Green- and red-only channels for GFAP and Olig2 staining, respectively, at 28 days post-insertion. White arrows denote GFAP+ (left) or Olig2+ (right) cells. Center of the probe hole is denoted by a white “x.” Scale bar = 50 μm. **(C)** Percent of GFAP+Olig2+ cells over total Olig2+ cells within 50 μm bins up to 300 μm away from site of insertion suggest preference for Olig2+GFAP+ cells to reside near site of probe implantation. ^∗^ indicates *p* < 0.05.

In order to evaluate if oligodendrocyte progenitors near the implant were differentiating into astrocytes, GFAP was co-labeled with the oligodendrocyte transcription factor Olig2. A subpopulation of Olig2+ astrocytes was observed around the implanted microelectrode array at 1, 3, 7, and 28 days post-insertion (Figure 5A). GFAP+ astrocytes co-localized with Olig2+ cells proximal to the site of insertion (Figure 5B). However, it is worth noting that Olig2+ staining appeared faint in GFAP+Olig2+ cells compared to GFAP-Olig2+ cells, possibly due to astrocyte downregulation of the Olig2 transcription factor. After 3 days post-insertion, the percentage of GFAP+Olig2+ cells out of the total Olig2+ population rose dramatically, significantly increasing between 0–50 μm away from the site of insertion at 7 and 28 days post-insertion and between 100–150 μm away from the site of insertion at 7 days post-insertion (*p* < 0.05) (Figure 5C and Supplementary Table S12). No significant difference in the percentage of GFAP+Olig2+ cells was noted between 7 and 28 days post-insertion at 100–150 μm away from the site of insertion. On day 1 post-insertion 0–50 μm away from the probe hole, there was an absence of GFAP+Olig2+ cells due to that fact that there was little to no remaining tissue within the 50 μm radius as well as reduced GFAP+ staining to begin with. At 7 and 28 days post-insertion, the percentage of GFAP+Olig2+ cells appeared elevated between 0–150 μm from the probe hole compared to distal regions, indicating a preference for localization close to the implanted device.

### PDGFRβ Immunoreactivity and Pattern of Blood-Brain Barrier Leakage Around Implanted Microelectrode Arrays

**FIGURE 6:**
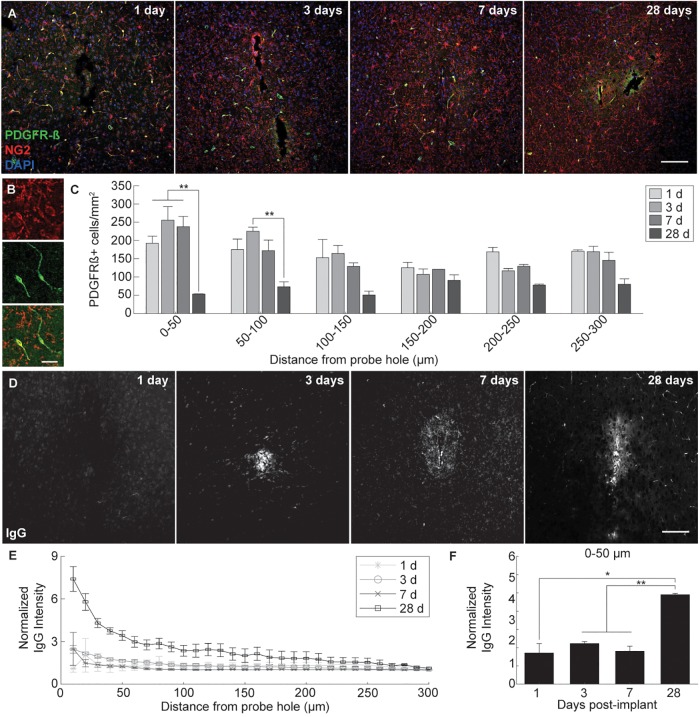
Pericyte dysregulation and blood-brain barrier disruption around an implanted microelectrode array. Microelectrode implantation induces pericyte reactivity and increased BBB leakage within close proximity to the electrode interface, suggesting a progressive dysregulation of the neurovascular unit following injury. **(A)** BBB-associated pericytes were visualized around implanted devices by representative immunohistochemical stains for PDGFR-β (green), NG2 (red), and cell nuclei (blue) at 1, 3, 7, and 28 days post-insertion. Scale bar = 100 μm. **(B)** Magnified images of NG2+ (top), PDGFR-β+ (middle), and NG2+PDGFR-β+ cells (bottom). Scale bar = 20 μm. **(C)** Spatiotemporal patterns of PDGFR-β+ cell density within 50 μm bins up to 300 μm away from the probe hole. **(D)** Temporal patterns of device-induced bleeding revealed by representative immunohistochemical stains for immunoglobulin G at 1, 3, 7, and 28 days post-insertion. **(E)** Normalized IgG intensity within 10 μm bins up to 300 μm away from the probe hole. **(F)** Normalized IgG intensity averaged over the first 50 μm from the probe hole. Increased IgG expression around the probe hole coincides with reduced pericyte densities at 28 days post-insertion. ^∗^ indicates *p* < 0.05. ^∗∗^ indicates *p* < 0.01.

**FIGURE 7:**
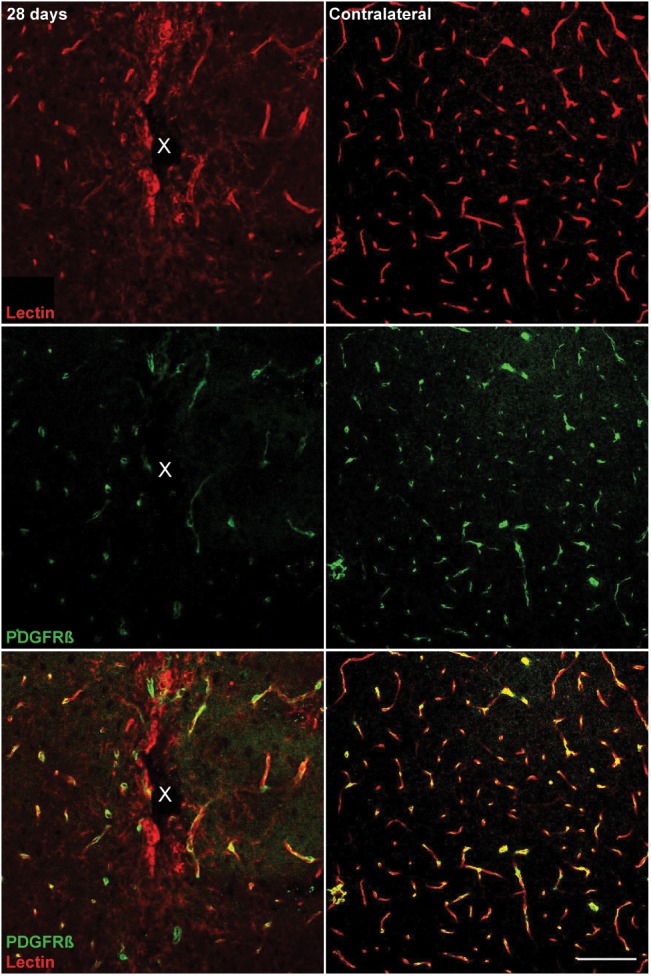
Reduced vascular structures around implanted microelectrode arrays at 28 days post-implantation. Chronic microelectrode implantation reveals specific insult to the endothelial component of the BBB, which may precede pericyte reactivity, vessel permeability, and leakage of plasma components. Blood vessels as well as vessel-bound pericytes are visualized by representative immunohistochemical stains for lectin (red) and PDGFR-β (green) at 28 days post-implantation compared to contralateral (non-implant) side. Center of the probe hole is denoted by a white “x.” Scale bar = 100 μm.

Microelectrode induced damage specific to the BBB was evaluated by tracking NG2+ perivascular pericytes at 1, 3, 7, and 28 days post-insertion (Figure 6A). Pericytes were identified from other NG2+ cells by co-localizing with platelet-derived growth factor receptor β (PDGFR-β), which is absent on NG2+ oligodendrocyte precursor cells (Figure 6B). Cell density analysis revealed an initial increase in PDGFR-β+ cells at 3 and 7 days post-insertion, most notably within a 50 μm radius from the site of insertion (Figure 6C and Supplementary Table S13). By 28 days post-insertion, the density of PDGFR-β+ cells was dramatically decreased around the microelectrode array and significantly different from PDGFR-β+ cell density at 1, 3, and 7 days post-insertion up to 50 μm away from the surface of the probe (*p* < 0.01). Coincidentally, analysis of BBB leakage via immunoglobulin G (IgG) staining revealed a temporal pattern of vascular disruption similar to the observed pericyte reactivity (Figure 6D). IgG fluorescence intensity was increased with proximity to the device for all time points, up to 50 μm away from the probe hole at 1, 3, and 7 days post-insertion, and up to 100–150 μm away from the probe hole at 28 days post-insertion (Figure 6E). Within the 50 μm region directly adjacent to the probe hole, IgG intensity was highest at 28 days post-insertion, significantly more increased than at 1, 3, and 7 days post-insertion (*p* < 0.05) (Figure 6F and Supplementary Table S14). Concurrent with reduced PDGFR-β immunoreactivity at chronic time points was a decrease in vascular structures around the implanted device. Tomato lectin staining demonstrated reduced blood vessel distribution around the site of implantation at 28 days post-insertion compared to the contralateral (non-implant) side (Figure 7).

## Discussion

The goal of this study was to evaluate novel histological markers around implanted intracortical probes in order provide further insight on dynamic tissue responses responsible for declining device performances. A more complete basic science knowledge of the tissue response to brain implants may facilitate the identification of novel opportunities for intervention strategies. It is understood that the homeostatic balance of the brain is not governed solely by neurons, microglia, and astrocytes, common targets while studying microelectrode-induced inflammation and injury. Similar to neurons, oligodendrocytes are metabolically active cells which can be considered just as important to brain circuit health given that they provide neurotrophic support and facilitate neuronal signaling via myelin sheath extension (Du and Dreyfus, 2002). Demyelination is a common symptom in many neurodegenerative diseases such as MS, stroke, and dementia, highlighting the susceptibility of oligodendrocytes to injury and their importance for neuronal survival (Dewar et al., 2003; Dulamea, 2017b). Additionally, oligodendrocyte precursors are versatile cells within the parenchyma, restoring oligodendrocyte populations following injury but are known to contribute to injury-induced inflammation (Dimou and Gallo, 2015). Finally, juxtavascular pericytes have direct associations with BBB health and are an understudied cell type around implanted intracortical devices, despite evidence that they also are implicated in a variety of neurodegenerative disorders (Winkler et al., 2011; Sweeney et al., 2016). Each of these CNS factors play either a direct or indirect role in regulating neuronal homeostasis and their respective dysfunctions following device implantation can affect critical neural circuits, altering recording or stimulating performances of intracortical electrode arrays.

### Neuronal Loss and Axonal Structural Changes Following Microelectrode Implantation

Distribution of neuronal cell bodies and axonal organization are significant factors around implanted microelectrode devices considering they are the electrically excitable components of the brain (Eles et al., 2018a). Neuronal cell density was disrupted mostly within the 0–50 μm region from the site of insertion. Within this radius, neuronal densities at 3 and 7 days post-insertion were slightly increased, which could be the result of fluctuations in tissue swelling and displacement around the device. Additionally, alterations in neurofilament intensity matched neural density changes within 50 μm from the probe hole at 1, 3, 7, and 28 days following implantation. Between 1 and 3 days post-insertion, neural density and neurofilament expression increase within 50 μm from the site of implantation. By 28 days post-insertion, a decrease in neurofilament expression within 50 μm away from the probe hole was observed coinciding with a drop in neural density. Gradually, the amount of caspase-3+ neurons increases from 1 to 28 days post-insertion, particularly within the 50 μm region around the device, indicating an increased propensity for these neurons near the device to undergo apoptotic cell death in response to the chronic implantation of a microelectrode array. Both neuronal density and axon neurofilament expression did not return to baseline levels until ∼150 μm away from the site of implantation. Considering that the maximum recordable radius of electrically active cells resides within this 150 μm radius (Buzsáki, 2004), changes to the organization of neurons and axons within this region may significantly influence the quality of device recording performance. However, the exact mechanisms governing this insult on neuronal viability remains unknown (Michelson et al., 2018b).

### Microelectrode Implantation Induces Spatial and Temporal Patterns Glial Cell Reactivity and Proliferation

Formation of a glial scar is another significant response to the implantation of a microelectrode array. Microglia and astrocyte glial membranes form a physical barrier between the electrode and neural tissue, which is understood to increase device impedances and directly alter the quality of the recorded signal (Williams et al., 2007; Alba et al., 2015). With specific regard to stimulating devices, gliosis has the potential to increase impedances, alter the material and mechanical properties at the tissue-electrode interface, and widen the distance between the device and the nearest active neuron, requiring higher stimulation thresholds which can pose even further complications to the tissue (Grill and Reichert, 2008; Kozai et al., 2014a; Thomas and Jobst, 2015; Campbell and Wu, 2018). Unlike recording performance, knowledge of stimulation performance suffers from additional parameter spaces and additional variability in performance outcomes (Platia and Brinker, 1986; Mushahwar et al., 2000; Branner et al., 2004; McCreery et al., 2010; Koivuniemi et al., 2011; Gittis, 2018). Reduction in signal-to-noise ratios can be attributed to increases in extracellular noise, which can occur due to the dysregulation of local ionic environments following glial activation. Furthermore, glial-secreted factors that promote inflammation and cell death can compromise neuronal or oligodendrocyte viability and exacerbate further glial activation and BBB disruption around the device (Biran et al., 2005; Karumbaiah et al., 2012; Prasad et al., 2012, 2014). Therapeutic approaches often attempt to attenuate glial cell activation or scar formation around implanted devices; however, reducing glial cell responses following nervous system injury carries the ability to worsen tissue health (Anderson et al., 2016). Therefore, future analyses focused on understanding glial cell dynamics after injury and their role during inflammation will help better understand wound healing and repair around implanted intracortical devices. Here, Iba-1+ microglia, NG2+ glia, and GFAP+ astrocyte fluorescence intensities were upregulated at different time points around implanted microelectrodes, indicating a temporal pattern of glial cell reactivity during device-induced inflammation.

Microglia are typically the first responders to injury within the CNS, their primary goal being to protect and repair injured tissue (Kawabori and Yenari, 2015). *In vivo* imaging has previously revealed that microglia respond immediately to the implantation of a microelectrode probe (Kozai et al., 2012a, 2016b; Eles et al., 2017). Following the initial response to electrode insertion, microglia begin migrating toward the device after 12 h of insertion (Kozai et al., 2016b; Wellman and Kozai, 2018). Here, Iba-1+ analysis indicated that microglia cells were the most significantly dividing glial cell type at 1 and 3 days post-insertion and that Iba-1+ fluorescence expression steadily increased up to 28 days post-insertion. The activation of microglia is followed by secretion of pro-inflammatory factors such as monocyte chemotactic protein (MCP-1), which induces the recruitment and migration of other immune cells, and tumor necrosis factor-alpha (TNF-α), which induces further glial cell activation and promotes neuron cell death (Biran et al., 2005). However, microglia can also secrete a range of anti-inflammatory cytokines to facilitate wound repair (Cherry et al., 2014). The complex range of microglia activity remains the focus of rigorous research, but these phenotypes can vastly alter the way microglia are viewed as a reactive glial cell type following CNS injury (Ransohoff, 2016; Eles et al., 2018b; Golabchi et al., 2018).

### Generation of Heterogeneous Population of Reactive Astrocytes Following Microelectrode Implantation

Following 1 week post-insertion, astrocytes were the dominating proliferative cell type with significantly elevated GFAP expression, indicative of their characteristic encapsulation of intracortical devices during glial scar formation. Of the observed GFAP+ population around implanted microelectrode arrays, GFAP+Olig2+ cells appeared with increasing frequency both as time of implantation progressed and with close proximity to the site of device insertion. The Olig2 marker is an oligodendrocyte transcription factor expressed solely within the central nervous system, responsible for regulating oligodendrocyte differentiation (Zhou et al., 2001). It is a common marker used to identify oligodendrocytes and oligodendrocyte precursors. Olig2 is known to increase following stab wound injury in the cortical gray matter, and the proportion of GFAP+Olig2+ cells within the Olig2+ population was reported between 7–9% at 3 and 7 days post-injury (Buffo et al., 2005). In contrast, this study demonstrates that GFAP+Olig2+ cells make up about 35–45% of the Olig2+ cell population between 3 and 7 days post-insertion, most likely attributed to the chronic presence of an implanted device compared to a transient stab wound injury. Previous studies have suggested that NG2 glia maintain the ability to differentiate into reactive astrocytes following injury (Dimou and Gallo, 2015). Whether these GFAP+Olig2+ cells were previously NG2+Olig2+ oligodendrocyte precursor cells or a separate Olig2+ subpopulation poses an interesting question for tissue injury and repair.

Observing NG2 glia differentiation into astrocytes has previously proven challenging since the *Cspg4* promoter encoding for the NG2 antigen is downregulated during differentiation rendering co-localization using immunohistochemical markers difficult and therefore requires use of retroviral or transgenic manipulations. In the healthy postnatal cortex, it has been shown that local proliferation of astrocytes, not NG2 glia, account for a majority of the astrocyte population (Ge et al., 2012; Ge and Jia, 2016). Previous studies have used Cre-loxP transgenic animals to track the cell fate of NG2 glia following injury *in vivo*. Komitova et al. (2011) determined that NG2 glia are not a significant source of reactive astrocytes following stab wound injury to the cortex, declaring only 8% of reporter-labeled cells who are also labeled GFAP-positive at 10 days post-injury. However, a study conducted by Hackett et al. (2016) observed 25% of NG2 glia express GFAP 7 days following contusive spinal cord injury, indicating that the severity of injury can influence the potential for astrogliogenesis of NG2 glia. Furthermore, a prior study evaluating the extent of NG2-derived astrocytes using transgenic lineage tracing techniques in either spinal cord injury or experimental autoimmune encephalomyelitis (EAE) demonstrated an increased percentage of NG2-differentiated astrocytes in SCI (25–40%) compared to EAE (9%) 4 weeks following injury (Hackett et al., 2018). In the case of microelectrode implantation where inflammation and secondary damage persists due to the chronic presence of the implant, the conversion of NG2 glia into reactive astrocytes could be a major contributor to the gliosis observed around acute and chronically implanted neural probes. The relevance of this phenomenon depends on the origin of reactive astrocytes. For example, astrocytes proliferate readily following 1 week after a stab wound injury to the cortex (Allahyari and Garcia, 2015). This presents two possible sources of origin for reactive astrocytes following device implantation: (1) from existing astrocytes which divide and migrate toward the electrode or (2) from NG2 glia which either (i) differentiate into astrocytes, (ii) proliferate and then differentiate into astrocytes, or (iii) both. Interestingly, NG2 glia intensity and proliferation decreases while astrocyte reactivity and proliferation peaks at 7 days post-insertion, suggesting a transformation of NG2 glia into reactive astrocytes following device implantation. However, the precise contribution from either source remains to be determined and may alter the focus of future research investigating the nature of scar formation following injury.

### Robustness of Oligodendrocyte and Oligodendrocyte Precursor Population Following Intracortical Microelectrode Implantation

Oligodendrocyte densities around the microelectrode device revealed a different pattern compared to neuronal distributions. These cells appeared more resistant to the acute and chronic implantation of a microelectrode device compared to neurons, remaining relatively stable in density throughout the time course of implantation. However, oligodendrocyte density was slightly decreased at 3 and 7 days post-insertion. Caspase-3 analysis revealed that oligodendrocyte dysfunction steadily increased up to 7 days post-insertion before decreasing by 28 days. Furthermore, degenerated oligodendrocytes appeared within close proximity to the microelectrode array, indicating a device-induced mechanism of cell death similar to neurons. By 28 days post-insertion, oligodendrocyte densities returned, coinciding with a decrease in apoptosis. Concomitantly, myelin basic protein expression was elevated around the implanted device at 28 days following insertion, suggesting a potential attempt at regeneration of oligodendrocytes and myelination following implantation-induced inflammation and injury. Unlike neurons, oligodendrocytes are supported by a dense and reactive oligodendrocyte precursor population that proliferate and differentiate following injury in order to sustain oligodendrocyte cell numbers (Levine et al., 2001). In some demyelinating diseases such as multiple sclerosis, differentiation of these oligodendrocyte precursor cells is impaired, leading to decreased oligodendrocyte numbers coinciding with increasing neurodegenerative deficits (Dulamea, 2017a). While oligodendrocyte regeneration and remyelination following brain injury is observed, newly formed myelin sheaths can return thinner than normal (Ishii et al., 2012). Although this study did not quantify myelin sheath thickness, integration and health of newly regenerated oligodendrocytes and myelin around implanted devices should be investigated further.

NG2+ cells were most activated at 28 days post-insertion, demonstrating significant proliferation and fluorescent expression of the NG2 antigen around the microelectrode device. It is possible this increase in oligodendrocyte precursor cell population is an attempt to facilitate oligodendrocyte regeneration and remyelination chronically around the device. Pericytes also express the NG2 antigen; however, pericyte reactivity was reduced around chronically implanted microelectrode arrays, therefore, is unlikely that the increase in NG2+ cell proliferation and intensity expression at 28 days post-insertion can be attributed to the pericyte population. Even then, NG2, which is a chondroitin sulfate proteoglycan known for axon growth inhibition within the CNS, can be cleaved and secreted extracellularly, which is why NG2 fluorescence intensity profiles may not be a direct indicator of NG2 glia distribution or behavior. Additional analysis such as chronic *in vivo* experimentation would be needed to determine NG2 glia dynamics around implanted microelectrode arrays. Moreover, thoroughly understanding the behavior of this precursor population following injury will provide future insight on wound repair and regeneration following injury.

### Loss of PDGFR-β+ Reactivity and Increased Blood-Brain Barrier Leakage Around Chronically Implanted Microelectrode Arrays

BBB degradation following microelectrode insertion are more frequently becoming a factor in studies concerning biological responses to implanted devices (Bjornsson et al., 2006; Johnson et al., 2007; Kozai et al., 2010, 2015c; Saxena et al., 2013; Nolta et al., 2015; Bedell et al., 2018; Bennett et al., 2019). Insertion of a device through the BBB is inevitable and can be severe depending on the size of the vessel ruptured (Kozai et al., 2010). Bleeding and loss of perfusion due to BBB damage can be detrimental to the viability of metabolically dependent cells such as neurons and oligodendrocytes within the brain (Kozai et al., 2015c; Wellman and Kozai, 2017; Wellman et al., 2018; Baranov et al., 2019). Additionally, inflammatory plasma proteins exposed to the parenchyma and biofouling on the implant surface have the potential to promote glial cell activation and further BBB breakdown (Kozai et al., 2012a). Avoiding or protecting the vasculature during insertion and chronic implantation of a device may potentially improve device performances by reducing inflammation within the brain. However, BBB function is supported by a variety of cells within the brain and each individual component can be influenced separately and distinctly by the chronic implantation of an intracortical electrode array (Muoio et al., 2014). Pericytes, which are gatekeepers to large macromolecules and circulating cells between the brain and the periphery, have many functional roles concerning BBB maintenance (Sweeney et al., 2016). From a tissue regenerative perspective, pericytes can facilitate new vessel formation and BBB repair whereas, during injury, pericytes can demonstrate neuroinflammatory functions and have been implicated in glial scar formation (Sweeney et al., 2016).

In this study, PDGFR-β+ cells were increased within the vicinity of an implanted device at 3 and 7 days following insertion. This increase in pericyte density could be an attempt to repair damaged vessels or mediate angiogenesis around the implanted device. By 28 days post-insertion, PDGFR-β+ cells were dramatically decreased compared to acute implantation. Previously, pericyte deficiencies during injury have been correlated with increased BBB dysfunction (Montagne et al., 2018). Indeed, bleeding was noticeably increased around the implanted device by 28 days post-insertion. However, vascular structures also appeared altered at chronic time points, prompting the question about whether reduced pericyte density is a consequence or effector of BBB disruption around inserted devices. Pericytes are increasingly becoming major cellular targets in neurodegenerative diseases such as Alzheimer’s, stroke, MS, and more (Winkler et al., 2011; Sweeney et al., 2016; Iacobaeus et al., 2017; Cheng et al., 2018). Further characterization of their behavior around implanted intracortical devices will help understand BBB associated inflammation following injury (Kozai et al., 2014c, 2015c; Wellman and Kozai, 2017).

## Conclusion

Investigation of various aspects of biological inflammation revealed spatiotemporal patterns of cell death, glial proliferation, and BBB-associated pathology around implanted intracortical microelectrode arrays. Neuronal loss was prominent near the site of electrode insertion at acute and chronic time points, coinciding with structural changes to local axons, primarily in an apoptotic-dependent manner. Similarly, apoptosis-induced oligodendrocyte cell death was prevalent at the time of acute implantation prior to a tissue regenerative attempt at restoring oligodendrocyte densities and enhancing myelination at chronic time points. Activation and proliferation of microglia, astrocytes, and NG2 glia were observed preferentially around inserted devices at distinct time points along the course of implantation. Furthermore, a novel subtype of reactive astrocytes was revealed around the site of implantation, potentially derived from a resident oligodendrocyte precursor population. Finally, chronic pericyte deficiency was noted alongside increased vascular dysfunction near inserted devices. This study simultaneously broadens the scope of dynamic tissue events that occur during intracortical device implantation and specifies distinct mechanisms of cell death and reactivity in response to inflammation. Future studies investigating the biological response to electrode-induced injury and inflammation in an attempt to improve device performances will benefit within the context of this newly discovered knowledge.

## Ethics Statement

All procedures and experimental protocols were approved by the University of Pittsburgh, Division of Laboratory Animal Resources, and Institutional Animal Care and Use Committee in accordance with standards for the humane animal case as set by the Animal Welfare Act and the National Institutes of Health Guide for the Care and Use of Laboratory Animals.

## Author Contributions

SW and TK contributed to conceptualization of the manuscript and developed the original draft of the manuscript. SW and IM performed the experimental surgeries. LL performed the sectioning, staining, and imaging of experimental samples. SW and YY conducted the image processing and analyses of histological data. All authors contributed to review and final edits.

## Conflict of Interest Statement

The authors declare that the research was conducted in the absence of any commercial or financial relationships that could be construed as a potential conflict of interest.

## References

[B1] Alba N. A. Du Z. J. Catt K. A. Kozai T. D. Y. Cui X. T. (2015). In vivo electrochemical analysis of a PEDOT/MWCNT neural electrode coating. *Biosensors* 5 618–646. 10.3390/bios5040618 26473938 PMC4697137

[B2] Allahyari R. V. Garcia A. D. R. (2015). Triggering reactive gliosis in vivo by a forebrain stab injury. *J. Vis. Exp.* e52825. 10.3791/52825 26167674 PMC4544677

[B3] Anderson M. A. Burda J. E. Ren Y. Ao Y. O’Shea T. M. Kawaguchi R. (2016). Astrocyte scar formation aids central nervous system axon regeneration. *Nature* 532:195. 10.1038/nature17623 27027288 PMC5243141

[B4] Armulik A. Genové G. Mäe M. Nisancioglu M. H. Wallgard E. Niaudet C. (2010). Pericytes regulate the blood–brain barrier. *Nature* 468:557. 10.1038/nature09522 20944627

[B5] Baranov S. V. Baranova O. V. Yablonska S. Suofu Y. Vazquez A. L. Kozai T. D. Y. (2019). Mitochondria modulate programmed neuritic retraction. *J. Proc. Natl. Acad. Sci. U.S.A.* 116 650–659. 10.1073/pnas.1811021116 30584104 PMC6329959

[B6] Bedell H. W. Hermann J. K. Ravikumar M. Lin S. Rein A. Li X. (2018). Targeting CD14 on blood derived cells improves intracortical microelectrode performance. *Biomaterials* 163 163–173. 10.1016/j.biomaterials.2018.02.014 29471127 PMC5841759

[B7] Bennett C. Mohammed F. Álvarez-Ciara A. Nguyen M. A. Dietrich W. D. Rajguru S. M. (2019). Neuroinflammation, oxidative stress, and blood-brain barrier (BBB) disruption in acute Utah electrode array implants and the effect of deferoxamine as an iron chelator on acute foreign body response. *Biomaterials* 188 144–159. 10.1016/j.biomaterials.2018.09.040 30343257 PMC6300159

[B8] Biran R. Martin D. C. Tresco P. A. (2005). Neuronal cell loss accompanies the brain tissue response to chronically implanted silicon microelectrode arrays. *Exp. Neurol.* 195 115–126. 10.1016/j.expneurol.2005.04.020 16045910

[B9] Bjornsson C. S. Oh S. J. Al-Kofahi Y. A. Lim Y. J. Smith K. L. Turner J. N. (2006). Effects of insertion conditions on tissue strain and vascular damage during neuroprosthetic device insertion. *J. Neural Eng.* 3 196–207. 10.1088/1741-2560/3/3/002 16921203

[B10] Bradl M. Lassmann H. (2010). Oligodendrocytes: biology and pathology. *Acta Neuropathol.* 119 37–53. 10.1007/s00401-009-0601-5 19847447 PMC2799635

[B11] Branner A. Stein R. B. Fernandez E. Aoyagi Y. Normann R. A. (2004). Long-term stimulation and recording with a penetrating microelectrode array in cat sciatic nerve. *IEEE Trans. Biomed. Eng.* 51 146–157. 10.1109/TBME.2003.820321 14723504

[B12] Buffo A. Vosko M. R. Ertürk D. Hamann G. F. Jucker M. Rowitch D. (2005). Expression pattern of the transcription factor Olig2 in response to brain injuries: implications for neuronal repair. *Proc. Natl. Acad. Sci. U.S.A.* 102 18183–18188. 10.1073/pnas.0506535102 16330768 PMC1312388

[B13] Buzsáki G. (2004). Large-scale recording of neuronal ensembles. *Nat. Neurosci.* 7 446–451. 10.1038/nn1233 15114356

[B14] Campbell A. Wu C. (2018). Chronically implanted intracranial electrodes: tissue reaction and electrical changes. *Micromachines* 9:430. 10.3390/mi9090430 30424363 PMC6187588

[B15] Cheng J. Korte N. Nortley R. Sethi H. Tang Y. Attwell D. (2018). Targeting pericytes for therapeutic approaches to neurological disorders. *Acta Neuropathol.* 136 507–523. 10.1007/s00401-018-1893-0 30097696 PMC6132947

[B16] Cherry J. D. Olschowka J. A. O’Banion M. K. (2014). Neuroinflammation and M2 microglia: the good, the bad, and the inflamed. *J. Neuroinflam.* 11:98. 10.1186/1742-2094-11-98 24889886 PMC4060849

[B17] Cheung T. Nuño M. Hoffman M. Katz M. Kilbane C. Alterman R. (2013). Longitudinal impedance variability in patients with chronically implanted DBS devices. *Brain Stimul.* 6 746–751. 10.1016/j.brs.2013.03.010 23619246

[B18] Dewar D. Underhill S. M. Goldberg M. P. (2003). Oligodendrocytes and ischemic brain injury. *J. Cereb. Blood Flow Metab.* 23 263–274. 10.1097/01.wcb.0000053472.41007.f9 12621301

[B19] Dimou L. Gallo V. (2015). NG2-glia and their functions in the central nervous system. *Glia* 63 1429–1451. 10.1002/glia.22859 26010717 PMC4470768

[B20] Du Y. Dreyfus C. F. (2002). Oligodendrocytes as providers of growth factors. *J. Neurosci. Res.* 68 647–654. 10.1002/jnr.10245 12111826

[B21] Du Z. J. Kolarcik C. L. Kozai T. D. Y. Luebben S. D. Sapp S. A. Zheng X. S. (2017). Ultrasoft microwire neural electrodes improve chronic tissue integration. *Acta Biomater.* 53 46–58. 10.1016/j.actbio.2017.02.010 28185910 PMC5512864

[B22] Dulamea A. O. (2017a). “Role of Oligodendrocyte Dysfunction in Demyelination, Remyelination and Neurodegeneration in Multiple Sclerosis,” in *Multiple Sclerosis: Bench to Bedside: Global Perspectives on a Silent Killer*, eds Asea A. A. A. Geraci F. Kaur P. (Cham: Springer International Publishing), 91–127. 10.1007/978-3-319-47861-6_7 28093710

[B23] Dulamea A. O. (2017b). The contribution of oligodendrocytes and oligodendrocyte progenitor cells to central nervous system repair in multiple sclerosis: perspectives for remyelination therapeutic strategies. *Neural Regenerat. Res.* 12 1939–1944. 10.4103/1673-5374.221146 29323026 PMC5784335

[B24] Eles J. Vazquez A. Kozai T. Cui X. (2018a). In vivo imaging of neuronal calcium during electrode implantation: spatial and temporal mapping of damage and recovery. *Biomaterials* 174 79–94. 10.1016/j.biomaterials.2018.04.043 29783119 PMC5987772

[B25] Eles J. Vazquez A. Kozai T. Cui X. T. (2018b). Meningeal inflammatory response and fibrous tissue remodeling around intracortical implants: an in vivo two-photon imaging study. *Biomaterials* 195 111–123. 10.1016/j.biomaterials.2018.12.031 30634095 PMC6350934

[B26] Eles J. R. Vazquez A. L. Snyder N. R. Lagenaur C. Murphy M. C. Kozai T. D. Y. (2017). Neuroadhesive L1 coating attenuates acute microglial attachment to neural electrodes as revealed by live two-photon microscopy. *Biomaterials* 113 279–292. 10.1016/j.biomaterials.2016.10.054 27837661 PMC5563378

[B27] Ge W. P. Jia J. M. (2016). Local production of astrocytes in the cerebral cortex. *Neuroscience* 323 3–9. 10.1016/j.neuroscience.2015.08.057 26343293 PMC4779064

[B28] Ge W.-P. Miyawaki A. Gage F. H. Jan Y. N. Janm L. Y. (2012). Local generation of glia is a major astrocyte source in postnatal cortex. *Nature* 484:376. 10.1038/nature10959 22456708 PMC3777276

[B29] Gittis A. (2018). Probing new targets for movement disorders. *Science* 361:462. 10.1126/science.aau4916 30072532

[B30] Golabchi A. Wu B. Li X. Carlisle D. L. Kozai T. D. Y. Friedlander R. M. (2018). Melatonin improves quality and longevity of chronic neural recording. *Biomaterials* 180 225–239. 10.1016/j.biomaterials.2018.07.026 30053658 PMC6179369

[B31] Grill W. M. Reichert W. (2008). “Signal considerations for chronically implanted electrodes for brain interfacing,” in *Indwelling Neural Implants: Strategies for Contending With the in Vivo Environment*, ed. Reichert W. (Boca Raton FL: CRC Press/Taylor & Francis), 42–58. 21204401

[B32] Hackett A. R. D-Lee H. Dawood A. Rodriguez M. Funk L. Tsoulfas P. (2016). STAT3 and SOCS3 regulate NG2 cell proliferation and differentiation after contusive spinal cord injury. *Neurobiol. Dis.* 89 10–22. 10.1016/j.nbd.2016.01.017 26804026 PMC4785033

[B33] Hackett A. R. Yahn S. L. Lyapichev K. Dajnoki A. D-Lee H. Rodriguez M. (2018). Injury type-dependent differentiation of NG2 glia into heterogeneous astrocytes. *Exp. Neurol.* 308 72–79. 10.1016/j.expneurol.2018.07.001 30008424 PMC6704012

[B34] Iacobaeus E. Sugars R. V. Törnqvist Andrén A. Alm J. J. Qian H. Frantzen J. (2017). Dynamic changes in brain mesenchymal perivascular cells associate with multiple sclerosis disease duration, active inflammation, and demyelination. *Stem Cells Transl. Med.* 6 1840–1851. 10.1002/sctm.17-0028 28941240 PMC6430046

[B35] Iordanova B. Vazquez A. L. Kozai T. D. Y. Fukuda M. Kim S. G. (2018). Optogenetic investigation of the variable neurovascular coupling along the interhemispheric circuits. *J. Cereb. Blood Flow Metab.* 38 627–640. 10.1177/0271678x18755225 29372655 PMC5888863

[B36] Ishii A. Fyffe-Maricich S. L. Furusho M. Miller R. H. Bansal R. (2012). ERK1/ERK2 MAPK signaling is required to increase myelin thickness independent of oligodendrocyte differentiation and initiation of myelination. *J. Neurosci.* 32 8855–8864. 10.1523/JNEUROSCI.0137-12.2012 22745486 PMC3521511

[B37] Johnson M. D. Kao O. E. Kipke D. R. (2007). Spatiotemporal pH dynamics following insertion of neural microelectrode arrays. *J. Neurosci. Methods* 160 276–287. 10.1016/j.jneumeth.2006.09.023 17084461

[B38] Karumbaiah L. Norman S. E. Rajan N. B. Anand S. Saxena T. Betancur M. (2012). The upregulation of specific interleukin (IL) receptor antagonists and paradoxical enhancement of neuronal apoptosis due to electrode induced strain and brain micromotion. *Biomaterials* 33 5983–5996. 10.1016/j.biomaterials.2012.05.021 22681976

[B39] Kawabori M. Yenari M. A. (2015). The role of the microglia in acute CNS injury. *Metab. Brain Dis.* 30 381–392. 10.1007/s11011-014-9531-6 24682762 PMC4180000

[B40] Koivuniemi A. Wilks S. J. Woolley A. J. Otto K. J. (2011). Multimodal, longitudinal assessment of intracortical microstimulation. *Progr. Brain Res.* 194 131–144. 10.1016/B978-0-444-53815-4.00011-X 21867800 PMC8098704

[B41] Komitova M. Serwanski D. R. Richard Lu Q. Nishiyama A. (2011). NG2 cells are not a major source of reactive astrocytes after neocortical stab wound injury. *Glia* 59 800–809. 10.1002/glia.21152 21351161 PMC3560299

[B42] Kozai T. Marzullo T. Hooi F. Langhals N. Majewska A. Brown E. (2010). Reduction of neurovascular damage resulting from microelectrode insertion into the cerebral cortex using in vivo two-photon mapping. *J. Neural Eng.* 7:046011. 10.1088/1741-2560/7/4/046011 20644246 PMC3164482

[B43] Kozai T. D. Y. Alba N. A. Zhang H. Kotov N. A. Gaunt R. A. Cui X. T. (2014a). “Nanostructured Coatings for Improved Charge Delivery to Neurons,” in *Nanotechnology and Neuroscience: Nano-electronic, Photonic and Mechanical Neuronal Interfacing*, eds De Vittorio M. Martiradonna L. Assad J. (New York, NY: Springer), 71–134. 10.1007/978-1-4899-8038-0_4

[B44] Kozai T. D. Y. Gugel Z. Li X. Gilgunn P. J. Khilwani R. Ozdoganlar O. B. (2014b). Chronic tissue response to carboxymethyl cellulose based dissolvable insertion needle for ultra-small neural probes. *Biomaterials* 35 9255–9268. 10.1016/j.biomaterials.2014.07.039 25128375

[B45] Kozai T. D. Y. Li X. Bodily L. M. Caparosa E. M. Zenonos G. A. Carlisle D. L. (2014c). Effects of caspase-1 knockout on chronic neural recording quality and longevity: Insight into cellular and molecular mechanisms of the reactive tissue response. *Biomaterials* 35 9620–9634. 10.1016/j.biomaterials.2014.08.006 25176060 PMC4174599

[B46] Kozai T. D. Y. Catt K. Li X. Gugel Z. V. Olafsson V. T. Vazquez A. L. (2015a). Mechanical failure modes of chronically implanted planar silicon-based neural probes for laminar recording. *Biomaterials* 37 25–39. 10.1016/j.biomaterials.2014.10.040 25453935 PMC4312222

[B47] Kozai T. D. Y. Du Z. Gugel Z. V. Smith M. A. Chase S. M. Bodily L. M. (2015b). Comprehensive chronic laminar single-unit, multi-unit, and local field potential recording performance with planar single shank electrode arrays. *J. Neurosci. Methods* 242 15–40. 10.1016/j.jneumeth.2014.12.010 25542351 PMC4432916

[B48] Kozai T. D. Y. Jaquins-Gerstl A. S. Vazquez A. L. Michael A. C. Cui X. T. (2015c). Brain tissue responses to neural implants impact signal sensitivity and intervention strategies. *ACS Chem. Neurosci.* 6 48–67. 10.1021/cn500256e 25546652 PMC4304489

[B49] Kozai T. D. Y. Catt K. Du Z. Na K. Srivannavit O. Haque R. M. (2016c). Chronic in vivo evaluation of PEDOT/CNT for stable neural recordings. *IEEE Trans. Biomed. Eng.* 63 111–119. 10.1109/TBME.2015.2445713 26087481 PMC4688254

[B50] Kozai T. D. Y. Eles J. R. Vazquez A. L. Cui X. T. (2016a). Two-photon imaging of chronically implanted neural electrodes: sealing methods and new insights. *J. Neurosci. Methods* 256 46–55. 10.1016/j.jneumeth.2015.10.007 26526459 PMC4771525

[B51] Kozai T. D. Y. Jaquins-gerstl A. S. Vazquez A. L. Michael A. C. Cui X. T. (2016b). Dexamethasone retrodialysis attenuates microglial response to implanted probes in vivo. *Biomaterials* 87 157–169. 10.1016/j.biomaterials.2016.02.013 26923363 PMC4866508

[B52] Kozai T. D. Y. Langhals N. B. Patel P. R. Deng X. Zhang H. Smith K. L. (2012a). Ultrasmall implantable composite microelectrodes with bioactive surfaces for chronic neural interfaces. *Nat. Mater.* 11 1065–1073. 10.1038/nmat3468 23142839 PMC3524530

[B53] Kozai T. D. Y. Vazquez A. L. Weaver C. L. Kim S.-G. Cui X. T. (2012b). In vivo two-photon microscopy reveals immediate microglial reaction to implantation of microelectrode through extension of processes. *J. Neural Eng.* 9:066001. 10.1088/1741-2560/9/6/066001 23075490 PMC3511663

[B54] Levine J. M. Reynolds R. Fawcett J. W. (2001). The oligodendrocyte precursor cell in health and disease. *Trends Neurosci.* 24 39–47. 10.1016/s0166-2236(00)01691-x 11163886

[B55] Lindahl P. Johansson B. R. Levéen P. Betsholtz C. (1997). Pericyte loss and microaneurysm formation in PDGF-B-deficient mice. *Science* 277 242–245. 10.1126/science.277.5323.242 9211853

[B56] McCreery D. Cogan S. Kane S. Pikov V. (2016). Correlations between histology and neuronal activity recorded by microelectrodes implanted chronically in the cerebral cortex. *J. Neural Eng.* 13:036012. 10.1088/1741-2560/13/3/036012 27108712 PMC7479968

[B57] McCreery D. Pikov V. Troyk P. R. (2010). Neuronal loss due to prolonged controlled-current stimulation with chronically implanted microelectrodes in the cat cerebral cortex. *J. Neural Eng.* 7:036005. 10.1088/1741-2560/7/3/036005 20460692 PMC2921317

[B58] McTigue D. M. Tripathi R. B. (2008). The life, death, and replacement of oligodendrocytes in the adult CNS. *J. Neurochem.* 107 1–19. 10.1111/j.1471-4159.2008.05570.x 18643793

[B59] Michelson N. J. Eles J. R. Vazquez A. L. Ludwig K. A. Kozai T. D. J. (2018a). Calcium activation of cortical neurons by continuous electrical stimulation: frequency dependence, temporal fidelity, and activation density. *J. Neurosci. Res.* 97 620–638. 10.1002/jnr.24370 30585651 PMC6469875

[B60] Michelson N. J. Vazquez A. L. Eles J. R. Salatino J. W. Purcell E. K. Williams J. J. (2018b). Multi-scale, multi-modal analysis uncovers complex relationship at the brain tissue-implant neural interface: new emphasis on the biological interface. *J. Neural Eng.* 15:033001. 10.1088/1741-2552/aa9dae 29182149 PMC5967409

[B61] Michelson N. J. Kozai T. D. Y. (2018). Isoflurane and ketamine differentially influence spontaneous and evoked laminar electrophysiology in mouse V1. *J. Neurophysiol.* 120 2232–2245. 10.1152/jn.00299.2018 30067128 PMC6295540

[B62] Montagne A. Nikolakopoulou A. M. Zhao Z. Sagare A. P. Si G. Lazic D. (2018). Pericyte degeneration causes white matter dysfunction in the mouse central nervous system. *Nat. Med.* 24:326. 10.1038/nm.4482 29400711 PMC5840035

[B63] Muoio V. Persson P. B. Sendeski M. M. (2014). The neurovascular unit – concept review. *Acta Physiol.* 210 790–798. 10.1111/apha.12250 24629161

[B64] Mushahwar V. K. Collins D. F. Prochazka A. (2000). Spinal cord microstimulation generates functional limb movements in chronically implanted cats. *Exp. Neurol.* 163 422–429. 10.1006/exnr.2000.7381 10833317

[B65] Nishiyama A. Yang Z. Butt A. (2005). Astrocytes and NG2-glia: what’s in a name? *J. Anat.* 207 687–693. 10.1111/j.1469-7580.2005.00489.x 16367796 PMC1571571

[B66] Nolta N. F. Christensen M. B. Crane P. D. Skousen J. L. Tresco P. A. (2015). BBB leakage, astrogliosis, and tissue loss correlate with silicon microelectrode array recording performance. *Biomaterials* 53 753–762. 10.1016/j.biomaterials.2015.02.081 25890770

[B67] Platia E. V. Brinker J. A. (1986). Time course of transvenous pacemaker stimulation impedance, capture threshold, and electrogram amplitude. *Pacing Clin. Electrophysiol.* 9 620–625. 10.1111/j.1540-8159.1986.tb05408.x 2429266

[B68] Polikov V. S. Tresco P. A. Reichert W. M. (2005). Response of brain tissue to chronically implanted neural electrodes. *J. Neurosci. Methods* 148 1–18. 10.1016/j.jneumeth.2005.08.015 16198003

[B69] Potter K. A. Buck A. C. Self W. K. Capadona J. R. (2012). Stab injury and device implantation within the brain results in inversely multiphasic neuroinflammatory and neurodegenerative responses. *J. Neural Eng.* 9:046020. 10.1088/1741-2560/9/4/046020 22832283

[B70] Prasad A. Xue Q.-S. Dieme R. Sankar V. Mayrand R. Nishida T. (2014). Abiotic-biotic characterization of Pt/Ir microelectrode arrays in chronic implants. *Front. Neuroeng.* 7:2. 10.3389/fneng.2014.00002 24550823 PMC3912984

[B71] Prasad A. Xue Q. S. Sankar V. Nishida T. Shaw G. Streit W. J. (2012). Comprehensive characterization and failure modes of tungsten microwire arrays in chronic neural implants. *J. Neural Eng.* 9:056015. 10.1088/1741-2560/9/5/056015 23010756

[B72] Ransohoff R. M. (2016). A polarizing question: do M1 and M2 microglia exist? *Nat. Neurosci*. 19:987. 10.1038/nn.4338 27459405

[B73] Salatino J. W. Ludwig K. A. Kozai T. D. Y. Purcell E. K. (2017). Glial responses to implanted electrodes in the brain. *Nature BME* 1 862–877. 10.1038/s41551-017-0154-1 30505625 PMC6261524

[B74] Saxena T. Karumbaiah L. Gaupp E. A. Patkar R. Patil K. Betancur M. (2013). The impact of chronic blood–brain barrier breach on intracortical electrode function. *Biomaterials* 34 4703–4713. 10.1016/j.biomaterials.2013.03.007 23562053

[B75] Schindelin J. Arganda-Carreras I. Frise E. Kaynig V. Longair M. Pietzsch T. (2012). Fiji: an open-source platform for biological-image analysis. *Nat. Methods* 9:676. 10.1038/nmeth.2019 22743772 PMC3855844

[B76] Schwartz A. B. Cui X. T. Weber D. J. Moran D. W. (2006). Brain-controlled interfaces: movement restoration with neural prosthetics. *Neuron* 52 205–220. 10.1016/j.neuron.2006.09.019 17015237

[B77] Sillay K. A. Chen J. C. Montgomery E. B. (2010). Long-term measurement of therapeutic electrode impedance in deep brain stimulation. *Neuromodulation* 13 195–200. 10.1111/j.1525-1403.2010.00275.x 21992832

[B78] Snaidero N. Simons M. (2017). The logistics of myelin biogenesis in the central nervous system. *Glia* 65 1021–1031. 10.1002/glia.23116 28168748

[B79] Stocking K. C. Vazquez A. L. Kozai T. D. Y. (2019). Intracortical neural stimulation with untethered, ultrasmall carbon fiber electrodes mediated by the photoelectric effect. *IEEE Trans. Biomed. Eng.* 1. 10.1109/TBME.2018.2889832 30605086

[B80] Sweeney M. D. Ayyadurai S. Zlokovic B. V. (2016). Pericytes of the neurovascular unit: key functions and signaling pathways. *Nat. Neurosci.* 19:771. 10.1038/nn.4288 27227366 PMC5745011

[B81] Szarowski D. H. Andersen M. D. Retterer S. Spence A. J. Isaacson M. Craighead H. G. (2003). Brain responses to micro-machined silicon devices. *Brain Res.* 983 23–35. 10.1016/s0006-8993(03)03023-3 12914963

[B82] Tan A. M. Zhang W. Levine J. M. (2005). NG2: a component of the glial scar that inhibits axon growth. *J. Anat.* 207 717–725. 10.1111/j.1469-7580.2005.00452.x 16367799 PMC1571583

[B83] Thomas G. P. Jobst B. C. (2015). Critical review of the responsive neurostimulator system for epilepsy. *Med. Dev.* 8:405. 10.2147/MDER.S62853 26491376 PMC4598207

[B84] Tomassy G. S. Berger D. R. Chen H. H. Kasthuri N. Hayworth K. J. Vercelli A. (2014). Distinct profiles of myelin distribution along single axons of pyramidal neurons in the neocortex. *Science* 344 319–324. 10.1126/science.1249766 24744380 PMC4122120

[B85] Tresco P. A. Winslow B. D. (2011). The challenge of integrating devices into the central nervous system. *Crit. Rev. Biomed. Eng.* 39 29–44. 10.1615/critrevbiomedeng.v39.i1.30 21488813

[B86] Wellman S. M. Cambi F. Kozai T. D. Y. (2018). The role of oligodendrocytes and their progenitors on neural interface technology: a novel perspective on tissue regeneration and repair. *Biomaterials* 183 200–217. 10.1016/j.biomaterials.2018.08.046 30172245 PMC6469877

[B87] Wellman S. M. Eles J. R. Ludwig K. A. Seymour J. P. Michelson N. J. McFadden W. E. (2017). A materials roadmap to functional neural interface design. *Adv. Funct. Mater.* 28:1701269. 10.1002/adfm.201701269 29805350 PMC5963731

[B88] Wellman S. M. Kozai T. D. Y. (2017). Understanding the inflammatory tissue reaction to brain implants to improve neurochemical sensing performance. *ACS Chem. Neurosci.* 8 2578–2582. 10.1021/acschemneuro.7b00403 29120167 PMC5944321

[B89] Wellman S. M. Kozai T. D. Y. (2018). Invivo spatiotemporal dynamics of NG2 glia activity caused by neural electrode implantation. *Biomaterials* 164 121–133. 10.1016/j.biomaterials.2018.02.037 29501892 PMC5951685

[B90] Williams J. C. Hippensteel J. A. Dilgen J. Shain W. Kipke D. R. (2007). Complex impedance spectroscopy for monitoring tissue responses to inserted neural implants. *J. Neural Eng.* 4 410–423. 10.1088/1741-2560/4/4/007 18057508

[B91] Winkler E. A. Bell R. D. Zlokovic B. V. (2011). Central nervous system pericytes in health and disease. *Nat. Neurosci.* 14:1398. 10.1038/nn.2946 22030551 PMC4020628

[B92] Winslow B. D. Christensen M. B. Yang W. K. Solzbacher F. Tresco P. A. (2010). A comparison of the tissue response to chronically implanted Parylene-C-coated and uncoated planar silicon microelectrode arrays in rat cortex. *Biomaterials* 31 9163–9172. 10.1016/j.biomaterials.2010.05.050 20561678 PMC12327938

[B93] Winslow B. D. Tresco P. A. (2010). Quantitative analysis of the tissue response to chronically implanted microwire electrodes in rat cortex. *Biomaterials* 31 1558–1567. 10.1016/j.biomaterials.2009.11.049 19963267

[B94] Zhou Q. Choi G. Anderson D. J. (2001). The bHLH transcription factor Olig2 promotes oligodendrocyte differentiation in collaboration with Nkx2. 2. *Neuron* 31 791–807. 10.1016/s0896-6273(01)00414-7 11567617

