# Supplementary material for: Revealing Spatial and Temporal Patterns of Cell Death, Glial Proliferation, and Blood-Brain Barrier Dysfunction Around Implanted Intracortical Neural Interfaces
Source: Front Neurosci. 2019 May 28;13:493. doi: 10.3389/fnins.2019.00493 (PMC6546924; doi:10.3389/fnins.2019.00493)
Supplement: Supplementary file 1 [file Table_1.DOCX]

Revealing spatial and temporal patterns of cell death, glial proliferation, and blood-brain barrier dysfunction around implanted intracortical neural interfaces

Steven M. Wellman^1,2^, Lehong Li^1^, Yalikun Yaxiaer^3^, Ingrid McNamara^1^, Takashi D.Y. Kozai^1,2,4,5,6^*

1. Department of Bioengineering, University of Pittsburgh, Pittsburgh, PA
2. Center for the Basis of Neural Cognition, Pittsburgh, PA
3. Eberly College of Science, Pennsylvania State University, University Park, PA
4. Center for Neuroscience, University of Pittsburgh, Pittsburgh, PA
5. McGowan Institute of Regenerative Medicine, University of Pittsburgh, Pittsburgh, PA
6. NeuroTech Center, University of Pittsburgh Brain Institute, Pittsburgh, PA

*Correspondence:

Takashi D.Y. Kozai

tdk18@pitt.edu

Keywords: Oligodendrocytes, NG2 glia, pericytes, tissue-electrode interface, neurodegeneration, gliosis, glial cell division, inflammation

Number of words: ~7,000 words

Number of figures: 22 (1 table, 7 figures, 14 supplementary tables)

**Supplemental Data**

|  | % of NeuN+Casp3+/Casp3+ | | | |
| --- | --- | --- | --- | --- |
| *p* | 1 dpi | 3 dpi | 7 dpi | 28 dpi |
| 1 dpi | X | 0.38907 | ***0.02361*** | ***0.04707*** |
| 3 dpi | 0.38907 | X | ***0.03649*** | ***0.04092*** |
| 7 dpi | ***0.02361*** | ***0.03649*** | X | 0.51283 |
| 28 dpi | ***0.04707*** | ***0.04092*** | 0.51283 | X |

**Supplementary Table 1.** Results of unequal variance t-test of percent of Caspase-3+ cells which are NeuN+ over 1, 3, 7, and 28 dpi (days post-insertion); significance *p<*0.05; *n* = 3 per time point. Significant p-values are bolded and italicized.

|  | % of NeuN+Casp3+/NeuN+ | | | | |
| --- | --- | --- | --- | --- | --- |
|  | *p* | 1 dpi | 3 dpi | 7 dpi | 28 dpi |
| 0-50 µm from probe hole | 1 dpi | X | 0.3798 | 0.21009 | ***0.04647*** |
|  | 3 dpi | 0.3798 | X | 0.1651 | ***0.03972*** |
|  | 7 dpi | 0.21009 | 0.1651 | X | 0.15226 |
|  | 28 dpi | ***0.04647*** | ***0.03972*** | 0.15226 | X |
| 50-100 µm from probe hole | 1 dpi | X | 0.4153 | 0.16691 | ***0.00142*** |
|  | 3 dpi | 0.4153 | X | 0.45188 | ***0.00224*** |
|  | 7 dpi | 0.16691 | 0.45188 | X | ***0.00094*** |
|  | 28 dpi | ***0.00142*** | ***0.00224*** | ***0.00094*** | X |
| 100-150 µm from probe hole | 1 dpi | X | 0.77421 | 0.27462 | ***0.00741*** |
|  | 3 dpi | 0.77421 | X | 0.24516 | ***0.01981*** |
|  | 7 dpi | 0.27462 | 0.24516 | X | 0.0867 |
|  | 28 dpi | ***0.00741*** | ***0.01981*** | 0.0867 | X |
| 150-200 µm from probe hole | 1 dpi | X | 0.28833 | 0.42265 | 0.08265 |
|  | 3 dpi | 0.28833 | X | 0.42957 | 0.09987 |
|  | 7 dpi | 0.42265 | 0.42957 | X | 0.09576 |
|  | 28 dpi | 0.08265 | 0.09987 | 0.09576 | X |
| 200-250 µm from probe hole | 1 dpi | X | N/A | 0.42265 | 0.34227 |
|  | 3 dpi | N/A | X | 0.42265 | 0.34227 |
|  | 7 dpi | 0.42265 | 0.42265 | X | 0.43298 |
|  | 28 dpi | 0.34227 | 0.34227 | 0.43298 | X |
| 250-300 µm from probe hole | 1 dpi | X | 0.42265 | 0.42265 | 0.32925 |
|  | 3 dpi | 0.42265 | X | 0.65287 | 0.45543 |
|  | 7 dpi | 0.42265 | 0.65287 | X | 0.37773 |
|  | 28 dpi | 0.32925 | 0.45543 | 0.37773 | X |

**Supplementary Table 2.** Results of unequal variance t-test of percent of NeuN+ cells which are Caspase-3+ within binned distances from the probe hole over 1, 3, 7, and 28 dpi (days post-insertion); significance *p<*0.05; *n* = 3 per time point. Significant p-values are bolded and italicized.

|  | Normalized NF-200 Intensity | | | |
| --- | --- | --- | --- | --- |
| *p* | 1 dpi | 3 dpi | 7 dpi | 28 dpi |
| 1 dpi | X | ***0.03037*** | 0.11 | 0.6288 |
| 3 dpi | ***0.03037*** | X | 0.52724 | 0.29303 |
| 7 dpi | 0.11 | 0.52724 | X | 0.19591 |
| 28 dpi | 0.6288 | 0.29303 | 0.19591 | X |

**Supplementary Table 3.** Results of unequal variance t-test of normalized NF-200 intensity within 50 μm from the probe hole over 1, 3, 7, and 28 dpi (days post-insertion); significance *p<*0.05; *n* = 3 per time point. Significant p-values are bolded and italicized.

|  | % of CC1+Casp3+/Casp3+ | | | |
| --- | --- | --- | --- | --- |
| *p* | 1 dpi | 3 dpi | 7 dpi | 28 dpi |
| 1 dpi | X | 0.05367 | ***6.9E-05*** | 0.32661 |
| 3 dpi | 0.05367 | X | ***0.00484*** | 0.15498 |
| 7 dpi | ***6.9E-05*** | ***0.00484*** | X | ***0.00166*** |
| 28 dpi | 0.32661 | 0.15498 | ***0.00166*** | X |

**Supplementary Table 4.** Results of unequal variance t-test of Caspase-3+ cells which are CC1+ over 1, 3, 7, and 28 dpi (days post-insertion); significance *p<*0.05; *n* = 3 per time point. Significant p-values are bolded and italicized.

|  | % of CC1+Casp3+/CC1+ | | | | |
| --- | --- | --- | --- | --- | --- |
|  | *p* | 1 dpi | 3 dpi | 7 dpi | 28 dpi |
| 0-50 µm from probe hole | 1 dpi | X | 0.78631 | ***0.00243*** | ***0.02382*** |
|  | 3 dpi | 0.78631 | X | ***0.00352*** | ***0.03259*** |
|  | 7 dpi | ***0.00243*** | ***0.00352*** | X | ***0.00669*** |
|  | 28 dpi | ***0.02382*** | ***0.03259*** | ***0.00669*** | X |
| 50-100 µm from probe hole | 1 dpi | X | 0.31115 | 0.22326 | 0.31115 |
|  | 3 dpi | 0.31115 | X | 0.33177 | N/A |
|  | 7 dpi | 0.22326 | 0.33177 | X | 0.33177 |
|  | 28 dpi | 0.31115 | N/A | 0.33177 | X |
| 100-150 µm from probe hole | 1 dpi | X | 0.42265 | 0.16062 | 0.20268 |
|  | 3 dpi | 0.42265 | X | 0.18378 | 0.26129 |
|  | 7 dpi | 0.16062 | 0.18378 | X | 0.43436 |
|  | 28 dpi | 0.20268 | 0.26129 | 0.43436 | X |
| 150-200 µm from probe hole | 1 dpi | X | 0.42265 | 0.84315 | 0.42265 |
|  | 3 dpi | 0.42265 | X | 0.42265 | N/A |
|  | 7 dpi | 0.84315 | 0.42265 | X | 0.42265 |
|  | 28 dpi | 0.42265 | N/A | 0.42265 | X |
| 200-250 µm from probe hole | 1 dpi | X | 0.83521 | 0.86912 | 0.48363 |
|  | 3 dpi | 0.83521 | X | 0.9479 | 0.45634 |
|  | 7 dpi | 0.86912 | 0.9479 | X | 0.40815 |
|  | 28 dpi | 0.48363 | 0.45634 | 0.40815 | X |
| 250-300 µm from probe hole | 1 dpi | X | 0.68954 | 0.42265 | 0.68954 |
|  | 3 dpi | 0.68954 | X | 0.42265 | 1 |
|  | 7 dpi | 0.42265 | 0.42265 | X | 0.42265 |
|  | 28 dpi | 0.68954 | 1 | 0.42265 | X |

**Supplementary Table 5.** Results of unequal variance t-test of percent of CC1+ cells which are Caspase-3+ within binned distances from the probe hole over 1, 3, 7, and 28 dpi (days post-insertion); significance *p<*0.05; *n* = 3 per time point. Significant p-values are bolded and italicized.

|  | Normalized GFAP Intensity | | | |
| --- | --- | --- | --- | --- |
| *p* | 1 dpi | 3 dpi | 7 dpi | 28 dpi |
| 1 dpi | X | ***0.04841*** | ***0.0048*** | ***0.01522*** |
| 3 dpi | ***0.04841*** | X | ***0.03137*** | 0.0627 |
| 7 dpi | ***0.0048*** | ***0.03137*** | X | 0.10993 |
| 28 dpi | ***0.01522*** | 0.0627 | 0.10993 | X |

**Supplementary Table 6.** Results of unequal variance t-test of normalized GFAP intensity within 50 μm from the probe hole over 1, 3, 7, and 28 dpi (days post-insertion); significance *p<*0.05; *n* = 3 per time point. Significant p-values are bolded and italicized.

|  | Normalized NG2 Intensity | | | |
| --- | --- | --- | --- | --- |
| *p* | 1 dpi | 3 dpi | 7 dpi | 28 dpi |
| 1 dpi | X | 0.67941 | 0.80048 | ***0.01007*** |
| 3 dpi | 0.67941 | X | 0.59192 | 0.11096 |
| 7 dpi | 0.80048 | 0.59192 | X | ***0.00796*** |
| 28 dpi | ***0.01007*** | 0.11096 | ***0.00796*** | X |

**Supplementary Table 7.** Results of unequal variance t-test of normalized NG2 intensity within 50 μm from the probe hole over 1, 3, 7, and 28 dpi (days post-insertion); significance *p<*0.05; *n* = 3 per time point. Significant p-values are bolded and italicized.

|  | % of Ki67+ cells | | | |
| --- | --- | --- | --- | --- |
|  | *p* | Iba-1 | GFAP | NG2 |
| 1 day post-insertion | Iba-1 | X | ***0.00024*** | ***0.047*** |
|  | GFAP | ***0.00024*** | X | 0.61426 |
|  | NG2 | ***0.047*** | 0.61426 | X |
| 3 days post-insertion | Iba-1 | X | 0.09198 | 0.22146 |
|  | GFAP | 0.09198 | X | 0.18231 |
|  | NG2 | 0.22146 | 0.18231 | X |
| 7 days post-insertion | Iba-1 | X | 0.14035 | 0.48728 |
|  | GFAP | 0.14035 | X | ***0.02162*** |
|  | NG2 | 0.48728 | ***0.02162*** | X |
| 28 days post-insertion | Iba-1 | X | 0.86529 | ***0.02943*** |
|  | GFAP | 0.86529 | X | ***0.02899*** |
|  | NG2 | ***0.02943*** | ***0.02899*** | X |

**Supplementary Table 8.** Results of unequal variance t-test of percent of Ki67+ cells that are Iba-1+, GFAP+, or NG2+ over 1, 3, 7, and 28 days post-insertion; significance *p<*0.05; *n* = 3 per time point. Significant p-values are bolded and italicized.

|  | % of Iba-1+Ki67+/Iba-1+ | | | | |
| --- | --- | --- | --- | --- | --- |
|  | *p* | 1 dpi | 3 dpi | 7 dpi | 28 dpi |
| 0-50 µm from probe hole | 1 dpi | X | 0.98216 | ***0.00331*** | ***0.04687*** |
|  | 3 dpi | 0.98216 | X | ***0.02895*** | 0.14576 |
|  | 7 dpi | ***0.00331*** | ***0.02895*** | X | ***0.04831*** |
|  | 28 dpi | ***0.04687*** | 0.14576 | ***0.04831*** | X |
| 50-100 µm from probe hole | 1 dpi | X | 0.19563 | 0.20295 | 0.42265 |
|  | 3 dpi | 0.19563 | X | 0.47149 | 0.26835 |
|  | 7 dpi | 0.20295 | 0.47149 | X | 0.46737 |
|  | 28 dpi | 0.42265 | 0.26835 | 0.46737 | X |
| 100-150 µm from probe hole | 1 dpi | X | 0.42265 | 0.42265 | 0.42265 |
|  | 3 dpi | 0.42265 | X | 0.85124 | 0.64665 |
|  | 7 dpi | 0.42265 | 0.85124 | X | 0.59175 |
|  | 28 dpi | 0.42265 | 0.64665 | 0.59175 | X |
| 150-200 µm from probe hole | 1 dpi | X | 0.38548 | 0.24173 | 0.19349 |
|  | 3 dpi | 0.38548 | X | 0.31544 | 0.19284 |
|  | 7 dpi | 0.24173 | 0.31544 | X | 0.42265 |
|  | 28 dpi | 0.19349 | 0.19284 | 0.42265 | X |
| 200-250 µm from probe hole | 1 dpi | X | 0.05494 | 0.05494 | 0.05494 |
|  | 3 dpi | 0.05494 | X | N/A | N/A |
|  | 7 dpi | 0.05494 | N/A | X | N/A |
|  | 28 dpi | 0.05494 | N/A | N/A | X |
| 250-300 µm from probe hole | 1 dpi | X | 0.19538 | 0.42265 | N/A |
|  | 3 dpi | 0.19538 | X | 0.50991 | 0.19538 |
|  | 7 dpi | 0.42265 | 0.50991 | X | 0.42265 |
|  | 28 dpi | N/A | 0.19538 | 0.42265 | X |

**Supplementary Table 9.** Results of unequal variance t-test of percent of Iba-1+ cells which are Ki67+ within binned distances from the probe hole over 1, 3, 7, and 28 dpi (days post-insertion); significance *p<*0.05; *n* = 3 per time point. Significant p-values are bolded and italicized.

|  | % of GFAP+Ki67+/GFAP+ | | | | |
| --- | --- | --- | --- | --- | --- |
|  | *p* | 1 dpi | 3 dpi | 7 dpi | 28 dpi |
| 0-50 µm from probe hole | 1 dpi | X | 0.44697 | ***0.00099*** | 0.17192 |
|  | 3 dpi | 0.44697 | X | ***0.01159*** | 0.3735 |
|  | 7 dpi | ***0.00099*** | ***0.01159*** | X | 0.05484 |
|  | 28 dpi | 0.17192 | 0.3735 | 0.05484 | X |
| 50-100 µm from probe hole | 1 dpi | X | N/A | 0.1873 | 0.42265 |
|  | 3 dpi | N/A | X | 0.1873 | 0.42265 |
|  | 7 dpi | 0.1873 | 0.1873 | X | 0.41 |
|  | 28 dpi | 0.42265 | 0.42265 | 0.41 | X |
| 100-150 µm from probe hole | 1 dpi | X | N/A | 0.08794 | N/A |
|  | 3 dpi | N/A | X | 0.08794 | N/A |
|  | 7 dpi | 0.08794 | 0.08794 | X | 0.08794 |
|  | 28 dpi | N/A | N/A | 0.08794 | X |
| 150-200 µm from probe hole | 1 dpi | X | 0.92958 | 0.9592 | 0.42265 |
|  | 3 dpi | 0.92958 | X | 0.95373 | 0.42265 |
|  | 7 dpi | 0.9592 | 0.95373 | X | 0.18883 |
|  | 28 dpi | 0.42265 | 0.42265 | 0.18883 | X |
| 200-250 µm from probe hole | 1 dpi | X | 0.81796 | 0.50806 | 0.94709 |
|  | 3 dpi | 0.81796 | X | 0.36699 | 0.77701 |
|  | 7 dpi | 0.50806 | 0.36699 | X | 0.56637 |
|  | 28 dpi | 0.94709 | 0.77701 | 0.56637 | X |
| 250-300 µm from probe hole | 1 dpi | X | 1 | 0.42265 | 0.42265 |
|  | 3 dpi | 1 | X | 0.42265 | 0.42265 |
|  | 7 dpi | 0.42265 | 0.42265 | X | N/A |
|  | 28 dpi | 0.42265 | 0.42265 | N/A | X |

**Supplementary Table 10.** Results of unequal variance t-test of percent of GFAP+ cells which are Ki67+ within binned distances from the probe hole over 1, 3, 7, and 28 dpi (days post-insertion); significance *p<*0.05; *n* = 3 per time point. Significant p-values are bolded and italicized.

|  | % of NG2+Ki67+/NG2+ | | | | |
| --- | --- | --- | --- | --- | --- |
|  | *p* | 1 dpi | 3 dpi | 7 dpi | 28 dpi |
| 0-50 µm from probe hole | 1 dpi | X | 0.0971 | 0.58409 | ***0.01306*** |
|  | 3 dpi | 0.0971 | X | 0.17067 | ***0.00459*** |
|  | 7 dpi | 0.58409 | 0.17067 | X | ***0.01546*** |
|  | 28 dpi | ***0.01306*** | ***0.00459*** | ***0.01546*** | X |
| 50-100 µm from probe hole | 1 dpi | X | N/A | N/A | 0.21997 |
|  | 3 dpi | N/A | X | N/A | 0.21997 |
|  | 7 dpi | N/A | N/A | X | 0.21997 |
|  | 28 dpi | 0.21997 | 0.21997 | 0.21997 | X |
| 100-150 µm from probe hole | 1 dpi | X | 0.93781 | 0.42265 | 0.06468 |
|  | 3 dpi | 0.93781 | X | 0.42265 | 0.07467 |
|  | 7 dpi | 0.42265 | 0.42265 | X | 0.06373 |
|  | 28 dpi | 0.06468 | 0.07467 | 0.06373 | X |
| 150-200 µm from probe hole | 1 dpi | X | 0.62093 | 0.9043 | 0.7123 |
|  | 3 dpi | 0.62093 | X | 0.47678 | 0.90111 |
|  | 7 dpi | 0.9043 | 0.47678 | X | 0.5919 |
|  | 28 dpi | 0.7123 | 0.90111 | 0.5919 | X |
| 200-250 µm from probe hole | 1 dpi | X | 0.42265 | 0.22107 | 0.10431 |
|  | 3 dpi | 0.42265 | X | 0.3055 | 0.12999 |
|  | 7 dpi | 0.22107 | 0.3055 | X | 0.23913 |
|  | 28 dpi | 0.10431 | 0.12999 | 0.23913 | X |
| 250-300 µm from probe hole | 1 dpi | X | 0.30356 | 0.42265 | 0.11964 |
|  | 3 dpi | 0.30356 | X | 0.65598 | 0.53541 |
|  | 7 dpi | 0.42265 | 0.65598 | X | 0.26379 |
|  | 28 dpi | 0.11964 | 0.53541 | 0.26379 | X |

**Supplementary Table 11.** Results of unequal variance t-test of percent of NG2+ cells which are Ki67+ within binned distances from the probe hole over 1, 3, 7, and 28 dpi (days post-insertion); significance *p<*0.05; *n* = 3 per time point. Significant p-values are bolded and italicized.

|  | % of GFAP+Olig2+/Olig2+ | | | | |
| --- | --- | --- | --- | --- | --- |
|  | *p* | 1 dpi | 3 dpi | 7 dpi | 28 dpi |
| 0-50 µm from probe hole | 1 dpi | X | 0.42265 | ***0.03901*** | 0.07045 |
|  | 3 dpi | 0.42265 | X | ***0.03729*** | ***0.01006*** |
|  | 7 dpi | ***0.03901*** | ***0.03729*** | X | 0.391 |
|  | 28 dpi | 0.07045 | ***0.01006*** | 0.391 | X |
| 50-100 µm from probe hole | 1 dpi | X | 0.3739 | 0.06181 | 0.09807 |
|  | 3 dpi | 0.3739 | X | ***0.01332*** | 0.05654 |
|  | 7 dpi | 0.06181 | ***0.01332*** | X | 0.42588 |
|  | 28 dpi | 0.09807 | 0.05654 | 0.42588 | X |
| 100-150 µm from probe hole | 1 dpi | X | 0.18976 | 0.78348 | 0.5846 |
|  | 3 dpi | 0.18976 | X | 0.40998 | 0.34041 |
|  | 7 dpi | 0.78348 | 0.40998 | X | 0.79811 |
|  | 28 dpi | 0.5846 | 0.34041 | 0.79811 | X |
| 150-200 µm from probe hole | 1 dpi | X | 0.10024 | 0.31058 | 0.3134 |
|  | 3 dpi | 0.10024 | X | 0.06789 | 0.64926 |
|  | 7 dpi | 0.31058 | 0.06789 | X | 0.09807 |
|  | 28 dpi | 0.3134 | 0.64926 | 0.09807 | X |
| 200-250 µm from probe hole | 1 dpi | X | 0.46438 | 0.38637 | 0.20441 |
|  | 3 dpi | 0.46438 | X | 0.28765 | 0.18617 |
|  | 7 dpi | 0.38637 | 0.28765 | X | 0.20788 |
|  | 28 dpi | 0.20441 | 0.18617 | 0.20788 | X |
| 250-300 µm from probe hole | 1 dpi | X | 0.51767 | 0.14245 | 0.60211 |
|  | 3 dpi | 0.51767 | X | 0.04262 | 0.98034 |
|  | 7 dpi | 0.14245 | 0.04262 | X | 0.12361 |
|  | 28 dpi | 0.60211 | 0.98034 | 0.12361 | X |

**Supplementary Table 12.** Results of unequal variance t-test of percent of Olig2+ cells which are GFAP+ within binned distances from the probe hole over 1, 3, 7, and 28 dpi (days post-insertion); significance *p<*0.05; *n* = 3 per time point. Significant p-values are bolded and italicized.

|  | PDGFRβ+ cell density | | | | |
| --- | --- | --- | --- | --- | --- |
|  | *p* | 1 dpi | 3 dpi | 7 dpi | 28 dpi |
| 0-50 µm from probe hole | 1 dpi | X | 0.817 | 0.9894 | ***0.0077*** |
|  | 3 dpi | 0.817 | X | 1 | ***0.0001*** |
|  | 7 dpi | 0.9894 | 1 | X | ***0.0002*** |
|  | 28 dpi | ***0.0077*** | ***0.0001*** | ***0.0002*** | X |
| 50-100 µm from probe hole | 1 dpi | X | 0.9702 | 1 | 0.1322 |
|  | 3 dpi | 0.9702 | X | 0.9507 | ***0.0027*** |
|  | 7 dpi | 1 | 0.9507 | X | 0.1598 |
|  | 28 dpi | 0.1322 | ***0.0027*** | 0.1598 | X |
| 100-150 µm from probe hole | 1 dpi | X | 1 | 1 | 0.1272 |
|  | 3 dpi | 1 | X | 0.9995 | 0.0549 |
|  | 7 dpi | 1 | 0.9995 | X | 0.498 |
|  | 28 dpi | 0.1272 | 0.0549 | 0.498 | X |
| 150-200 µm from probe hole | 1 dpi | X | 1 | 1 | 0.9996 |
|  | 3 dpi | 1 | X | 1 | 1 |
|  | 7 dpi | 1 | 1 | X | 1 |
|  | 28 dpi | 0.9996 | 1 | 1 | X |
| 200-250 µm from probe hole | 1 dpi | X | 0.958 | 0.9978 | 0.2574 |
|  | 3 dpi | 0.958 | X | 1 | 0.9983 |
|  | 7 dpi | 0.9978 | 1 | X | 0.9631 |
|  | 28 dpi | 0.2574 | 0.9983 | 0.9631 | X |
| 250-300 µm from probe hole | 1 dpi | X | 1 | 1 | 0.2659 |
|  | 3 dpi | 1 | X | 1 | 0.2873 |
|  | 7 dpi | 1 | 1 | X | 0.7838 |
|  | 28 dpi | 0.2659 | 0.2873 | 0.7838 | X |

**Supplementary Table 13.** Results of two-way ANOVA followed by post-hoc Tukey’s HSD test of PDGFRβ+ cell density within binned distances from the probe hole over 1, 3, 7, and 28 dpi (days post-insertion); degrees of freedom = 3, F-value = 33.44, significance *p<*0.05; *n* = 3 per time point. Significant p-values are bolded and italicized.

|  | Normalized IgG Intensity | | | |
| --- | --- | --- | --- | --- |
| *p* | 1 dpi | 3 dpi | 7 dpi | 28 dpi |
| 1 dpi | X | 0.42762 | 0.87993 | ***0.02574*** |
| 3 dpi | 0.42762 | X | 0.24784 | ***3.2E-05*** |
| 7 dpi | 0.87993 | 0.24784 | X | ***0.00846*** |
| 28 dpi | ***0.02574*** | ***3.2E-05*** | ***0.00846*** | X |

**Supplementary Table 14.** Results of unequal variance t-test of normalized IgG intensity within 50 μm from the probe hole over 1, 3, 7, and 28 dpi (days post-insertion); significance *p<*0.05; *n* = 3 per time point. Significant p-values are bolded and italicized.
